# Boron Carbon Oxynitride as a Novel Metal-Free Photocatalyst

**DOI:** 10.1186/s11671-021-03629-5

**Published:** 2021-12-11

**Authors:** Liang Cheng Chien, Chen Wei Chiang, Chou Chio Lao, Yung-I Lin, Hao-Wu Lin, Pei Yuin Keng

**Affiliations:** Department of Materials Science and Engineering, National Tsing Hua University, Hsinchu City, 30013 Taiwan

**Keywords:** Boron carbon oxynitride (BCNO), Hexagonal boron nitride (hBN), Graphene oxides, Boron nanomaterials, Photocatalysts, Metal-free, Structure–property, Semiconductor, Nanophosphor, Two-dimensional materials

## Abstract

Boron-based nanomaterials are emerging as non-toxic, earth-abundant (photo)electrocatalyst materials in solar energy conversion for the production of solar hydrogen fuel and environmental remediation. Boron carbon oxynitride (BCNO) is a quaternary semiconductor with electronic, optical, and physicochemical properties that can be tuned by varying the composition of boron, nitrogen, carbon, and oxygen. However, the relationship between BCNO's structure and -photocatalytic activity relationship has yet to be explored. We performed an in-depth spectroscopic analysis to elucidate the effect of using two different nitrogen precursors and the effect of annealing temperatures in the preparation of BCNO. BCNO nanodisks (*D* = 6.7 ± 1.1 nm) with turbostratic boron nitride diffraction patterns were prepared using guanidine hydrochloride as the nitrogen source precursor upon thermal annealing at 800°C. The X-ray photoelectron spectroscopy (XPS) surface elemental analysis of the BCNO nanodisks revealed the B, C, N, and O compositions to be 40.6%, 7.95%, 37.7%, and 13.8%, respectively. According to the solid-state ^11^B NMR analyses, the guanidine hydrochloride-derived BCNO nanodisks showed the formation of various tricoordinate BN_*x*_(OH)_3−*x*_ species, which also served as one of the photocatalytic active sites. The XRD and in-depth spectroscopic analyses corroborated the preparation of BCNO-doped hexagonal boron nitride nanodisks. In contrast, the BCNO annealed at 600 °C using melamine as the nitrogen precursor consisted of layered nanosheets composed of B, C, N, and O atoms covalently bonded in a honeycomb lattice as evidence by the XRD, XPS, and solid-state NMR analysis (^11^B and ^13^C) analyses. The XPS surface elemental composition of the melamine-derived BCNO layered structures consisted of a high carbon composition (75.1%) with a relatively low boron (5.24%) and nitrogen (7.27%) composition, which indicated the formation of BCNO-doped graphene oxides layered sheet structures. This series of melamine-derived BCNO-doped graphene oxide layered structures were found to exhibit the highest photocatalytic activity, exceeding the photocatalytic activity of graphitic carbon nitride. In this layered structure, the formation of the tetracoordinate BN_*x*_(OH)_3−*x*_(CO) species and the rich graphitic domains were proposed to play an important role in the photocatalytic activity of the BCNO-doped graphene oxides layered structures. The optical band gap energies were measured to be 5.7 eV and 4.2 eV for BCNO-doped hexagonal boron nitride nanodisks and BCNO-doped graphene oxides layered structures, respectively. Finally, BCNO exhibited an ultralong photoluminescence with an average decay lifetime of 1.58, 2.10, 5.18, and 8.14 µs for BGH01, BGH03, BMH01, BMH03, respectively. This study provides a novel metal-free photocatalytic system and provides the first structural analysis regarding the origin of BCNO-based photocatalyst.

**Supplementary Information:**

The online version contains supplementary material available at 10.1186/s11671-021-03629-5.

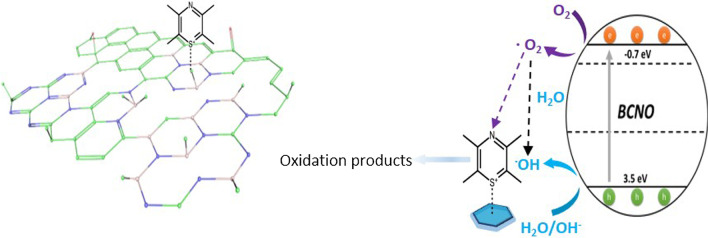

## Introduction

Metal-free nanomaterials are emerging as cost-effective, earth-friendly (photo)catalyst with high structural and chemical stabilities for various applications including solar fuel production, environmental remediation, CO_2_ reduction, disinfection of harmful microorganisms, and enabled selective chemical synthesis of organic compounds [1–7]. Compared to their metal counterparts, a metal-free catalyst is also less prone to poisoning and leads to higher cycle lifetimes. Thus, the search for and development of new materials that are stable, efficient, and cost-effective photocatalyst remains are critical and challenging research endeavors. Carbon-based materials such as graphitic carbon nitride (CN) [4, 8, 9], carbon-dot (C-dot) [2, 3, 10], and graphene-based materials [7, 11] have been widely investigated due to their excellent physicochemical properties, structural and chemical stabilities, and the ease of synthesis from earth-abundant elements. Recently, boron-based (photo)catalysts have been developed as metal-free photocatalytic systems with remarkable performance. Notably, boron carbide, known for its hardness, showed metal-free visible light photocatalytic hydrogen generation, surpassing the state-of-the-art carbon-based CN photocatalyst [12, 13]. High surface area carbon-doped hexagonal boron nitride (BCN) nanosheets exhibiting visible light photocatalytic activity for H_2_ andO_2_ generation as well as CO_2_ reduction and capture have led to new  possibilities in photosystem [14, 15]. Other boron-containing (photo)electrocatalyst such as boron oxynitride (BNO)[16, 17], boron phosphide (BP) [18, 19], boron-doped graphene [20], boron carbon nitride (BCN) [14], boron-doped carbon nitride (B-doped_CN_) [21] and elemental boron [22, 23] have demonstrated significant (photo) electrocatalytic activities [13].

Boron carbon oxynitride (BCNO) is a boron-based nanomaterials that has been studied less than other materials. It was first developed after their BCN predecessor, a semiconductor with a band gap of approximately 2 eV, to replace toxic phosphors based on oxynitride and nitride compounds [24, 25]. The substitution of B, C, O and N atom into the graphene or hexagonal boron nitride (hBN) network gave rise to BCNO compounds with tuneable photoluminescence properties and a bandgap ranging from 0 eV (graphene) to 5.9 eV (hBN) [26]. These desirable semiconduction and photoluminescence properties have recently attracted researchers to develop a new synthetic methodology to synthesize low dimensional BCNO nanostructures with higher crystallinity [27], controlled shapes [28], and in atomically thin 2D structures [29]. Previous works investigated the effects of annealing temperatures and times in modulating the photoluminescence property of BCNO without providing in-depth structural characterization [30, 31]. In this paper, we investigated the effect of using different nitrogen source precursors, calcination temperatures (800 °C vs. 600C), and calcination times (0.5 h vs. 12 h) on the structure-photocatalytic activity of BCNO nanostructures.

## Methods

### Chemicals and Instruments

Boric acid 99.99% (H_3_BO_3_), melamine 99% (C_3_H_6_N_6_), and hexamethylenetetramine ≥ 99% (C_6_H_12_N_4_) were purchased from Alfa Aesar and used without further purification. Guanidine hydrochloride 99.5% (CH_5_N_3_ HCl) was purchased from Arcos Organics. BCNO was synthesized according to the literature via the low-temperature annealing method [25, 32]. UPS analysis was performed in a ULVAC-PHI PHI 5000 Versaprobe II using He I 21.22 eV as a photon source with 5 V bias. The morphology of BCNO samples was analysed via a transmission electron microscope (JEOL, JEM-ARM200FTH). XRD diffractions were obtained using the Bruker D2 spectrometer. Photoluminescence emission spectra in solution were obtained using a photoluminescence spectrometer (PerkinElmer, LS55), and optical absorption spectra of BCNO in solution were determined by the UV–Vis spectrometer (HITACHI, U-3900). X-ray photoelectron spectroscopy was analysed via high-resolution X-ray photoelectron spectrometer (ULVAC-PHI, PHI Quantera II) using Al Ka x-rays as the excitation source. The BCNO solution was drop-casted onto the silicon substrate for XPS characterization. The binding energy was calibrated to carbon at 284.8 eV. XPS peak deconvolution and fitting were performed using CACS XPS software. Absolute PLQY was performed according to the literature [33] and detected by a CCD camera (PIXIS 256BR, Princeton Instruments). The measurement of absolute PLQY was performed by using a fiber-based spectrograph, including a calibrated integrating sphere system (Labsphere) and a charge-coupled device (CCD) camera (PIXIS 256BR, Princeton Instruments). A diode laser (*λ* = 375 nm, Becker & Hickl GmbH) was used as the pumping source. Time-resolved photoluminescence was measured in a front-face configuration by using a pulsed nitrogen laser (*λ* = 337.1 nm, LTB Lasertechnik Berlin GmbH) as an excitation source, which was triggered by a digital delay generator (DG645, Stanford Research Systems). The signals were detected by a photon-counting photomultiplier tube (PMC-100–1, Becker & Hickl GmbH), and the photon counts were accumulated with a multiscaler module (MSA-300, Becker & Hickl GmbH). Infrared spectra were recorded by Fourier-transform infrared spectrometer (Bruker, Vertex 80v) using Attenuated Total Reflectance (ATR). Nuclear magnetic resonance spectra were obtained using Bruker Avance III 400 NMR spectrometer equipped with a 9.4 T magnet using a 4 mm magic angle spinning (MAS) probe. ^11^B MAS NMR were recorded using the spin echo method with spinning rate of 10 kHz. 8000 scans were collected with 4 s recycle delay. Chemical shifts were referenced to 1 M H_3_BO_3_ aqueous solution at 19.6 ppm. ^13^C CP/MAS NMR spectra were recorded using a cross polarization (CP) sequence with spinning rate of 12.5 kHz. 30,000 scans were collected with 4 s recycle delay. All of the ^13^C chemical shifts were referenced to neat trimethylsilane using the secondary reference of the adamantane CH_2_ peak at 38.48 ppm.

### Preparation of BCNO

In this study, two series of BCNO were prepared using two different nitrogen precursor sources while fixing the boron and carbon precursor sources, precursor ratios, annealing temperature, and time. The effect of annealing temperatures and times were also investigated by fixing all the other reaction parameters for each series of BCNO prepared in this study. Briefly, all three precursor components in a predetermined mole ratio were added to distilled water and heated to 90 °C until the solution appeared homogeneous (Table 1). The glutinous mixture was dried in an oven overnight, yielding a dried white solid. The white solid was grounded using a mortar and pestle to fine powders. The solid precursor was calcined in the furnace at a predetermined temperature and time, as shown in Table 1, with a ramping rate of 5 °C/min under ambient atmospheric pressure. The yellowish-tinged powder sample was grounded to a fine powder after the furnace was naturally cooled down to room temperature.

**Table 1:** Precursor sources, ratios, annealing temperatures, and times for the preparation of BGH and BMH series

Precursor	Temperature	Time (h)	Mole ratio (B:N:C)	Sample name
1 (boric acid, guanidine hydrochloride, hexamethylenetetramine)	600 °C	12	3:1:0.1	BGH01LT
	800 °C	12	3:1:0.1	BGH01
	800 °C	12	3:1:0.3	BGH03
2 (boric acid, melamine, hexamethylenetetramine)	600 °C	12	3:1:0.1	BMH01
	800 °C	12	3:1:0.1	BMH01HT
	600 °C	12	3:1:0.3	BMH03

### Purification Strategies of BCNO

The as-prepared BCNO was purified by centrifugation at 6000 rpm for 10 min in water and ethanol (1:10 v/v in 10 mg/mL concentration). After centrifugation, the product was redissolved in distilled water, diluted with ethanol in a 1:10 v/v ratio of water: ethanol. The purified BCNO was deposited onto a carbon-coated copper grid for TEM analysis. For the preparation of SEM and XPS specimens, the purified BCNO sample was drop-casted onto a silicon wafer. Prior to sample deposition, silicon wafers were cleaned by sonication with water, propanol, and acetone for 10 min in each solvent. UPS specimen was prepared similarly to the procedure described for SEM and XPS samples, except that the samples were deposited onto an indium tin oxide (ITO) coated glass.

### Preparation of Bulk Carbon Nitride (CN)

Bulk CN was synthesized by a facile method reported in the literature [34]. Briefly, melamine powder was placed into a crucible and annealed at 550℃ for four hours with a ramping rate of 5 ℃/min under ambient atmospheric pressure.

### Photocatalytic Dye Degradation Procedure

The photocatalytic activities of various BCNO samples were evaluated via the methylene blue (MB) photodegradation as a model reaction. In a typical dye degradation experiment, 10 mg of BCNO sample was added into a sample vial containing 15 mL of MB solution (10 ppm). After stirring for 10 min in the dark, the sample vial was irradiated with a 100 W Xenon lamp (250 nm ~ 1100 nm). Kinetic samples (2 mL) were extracted using a pipette from the solution at 20 min time interval until the total photocatalytic degradation time reached 80 min. Kinetic samples at different time intervals were analysed via a UV–vis spectrometer. The change in the concentration of MB was derived using Beer's law.

## Results and Discussion

### Synthesis of BCNO

In our research for the preparation ofg low dimensional BCNO nanostructures with high crystallinity to facilitate charge transport, we discovered of BCNO with distinctly different chemical structures and photocatalytic activities. Based on the synthesis of BCNO reported in the literature [25, 32], we investigated the effect of using two different nitrogen precursor sources as well as the effect of thermal annealing temperatures, and time on the structure–property evolution of BCNO. In this study, two series of BCNO were prepared using boric acid and hexamethylenetetramine as boron and carbon sources, respectively (Table 1). The BCNO was synthesized using melamine and guanidine hydrochloride as the nitrogen source and is denoted as BMH and BGH, respectively. After  systematically investigating each reaction condition, the BMH series only exhibited photocatalytic activities when annealed at the lower temperature of 600 °C for 12 hr. The BGH series only exhibited photocatalytic activities with high-temperature annealing at 800 °C for 12 h. In both series, the mole ratios of boric acid, melamine, and guanidine hydrochloride were fixed at 3:1, while the mole ratios of hexamethylenetetramine were varied from 0.1 to 0.3. These ratiso of hexamethylenetetramine precursor are denoted in the sample names as BMHH01/BGH01 and BMH03/BGH03, respectively (Table 1).

**Fig. 1:**
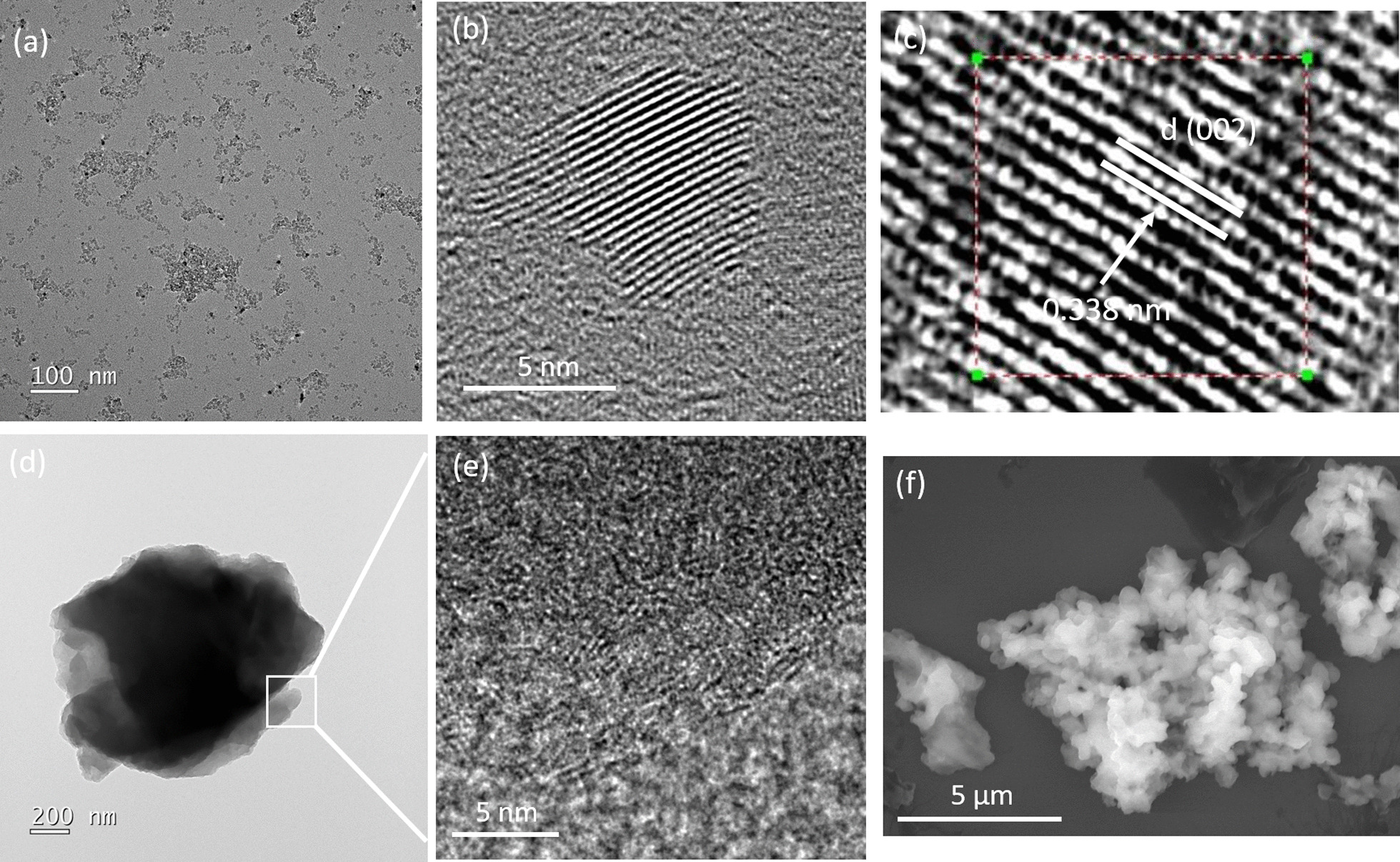
Representative transmission electron micrograph (TEM) and scanning electron micrograph (SEM) of the as-prepared BGH and BMH deposited from a dilute ethanol solution. **a**, **b** High and low magnification TEM of BGH, **c** high-resolution TEM of BGH with distinct (002) lattice spacing of 0.338 nm, **d** TEM image of representative BMH at low magnification, **e** expanded TEM image of the boxed area in Fig. 1**d** showing features of structural distortion within the layered graphene oxide, and **f** representative SEM image of BMH

Figure 1 shows the representative TEM images of BGH calcined at 800 °C for 12 h composed of a crystalline core with quasi-spherical nanoparticle morphology and *D* = 6.7 ± 1 nm. The as-prepared BGH was readily dispersed in water (Additional file 1: Fig. S1), and as evidenced by the deposition of discrete nanoparticles on the TEM copper grid. The aqueous dispersibility of BGH and BMH are most likely originated from electrostatic stabilization based on their negatively charged surface potential of BGH and BMH. Solid-state ^11^B NMR analyses revealed that the abundance of hydroxyl groups in both BMH and BGH nanostructures corroborated their dispersibility in aqueous media. Under high magnification, each quasi-spherical nanoparticle exhibited distinctive lattice spacing of the (002) planes measured to be 0.338 nm, which is consistent with literature reports [24, 35] (Fig. 1c). Compared to the 5 nm BCNO nanoparticle obtained through thermal annealing in the eutectic salt environment, the BGH nanoparticles prepared in this work possessed high crystallinity [25] (Fig. 1). Next, we characterized BMH that was prepared based on a literature report using boric acid, melamine, and hexamethylenetetramine and annealed at 600 °C for 12 h. In contrast to the morphology of BGH, BMH is composed of multi-layered sheets with ill-defined shapes (Fig. 1). At higher magnification, the TEM image of the edges on the multilayer sheets (Fig. 1d) revealed nanosheets with features of structural distortion [36] as shown in Fig. 1e. Figure 1f shows a representative SEM image of the BMH including featureless micron-sized aggregates. However, unlike the BGH series, higher temperature calcination at 800 °C using melamine as the nitrogen source precursor did not yield a nanodisk morphology. The TEM, XRD, UV absorbance, and photoluminescence of the other BGH and BMH series compounds synthesized at different reaction conditions are available in the ESI.

**Fig. 2:**
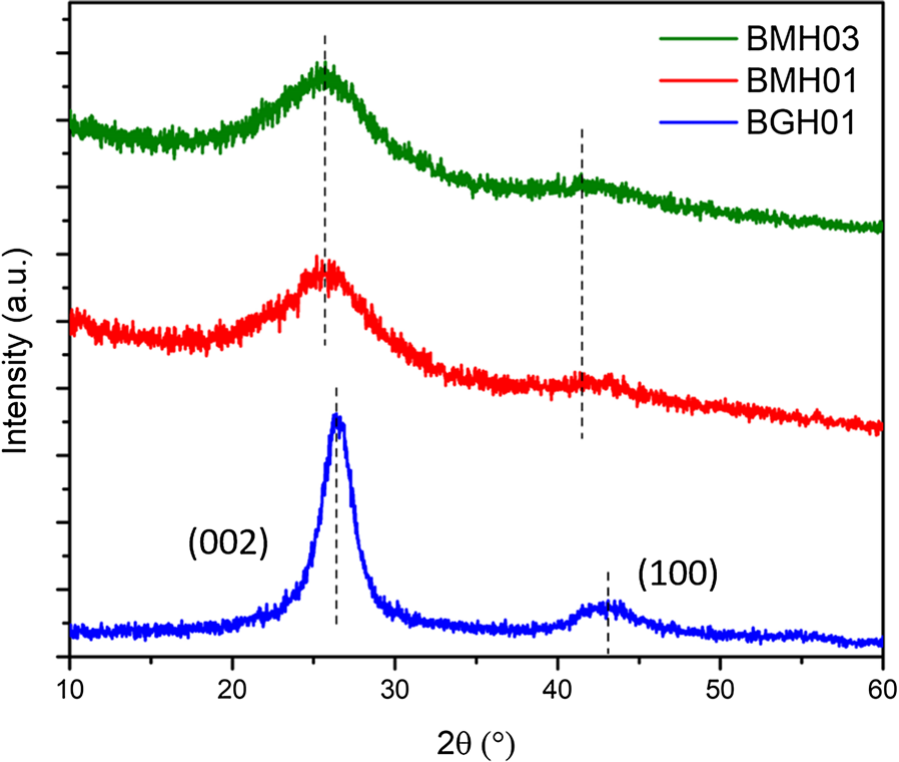
The XRD patterns of polycrystalline BMH03 (green trace), BMH01 (red trace), BGH01 (blue trace), and BGH01. The broad peak of the BGH01 series at ca. 26.6° represents the (002) plane reflection of h-BN and another broad peak at ca. 43.1° represents the unresolved reflection planes of h-BN. Diffraction patterns of the BMH showed two broad reflections centered at around ~ 25.4° and ~ 42.4°. The XRD patterns of other BCNO series are shown in Additional file 1: Figure S3)

Figure 2 shows the XRD patterns of BMH03 (green trace), BMH01 (red trace), and BGH01 (blue trace) prepared in our laboratory at the prescribed reaction conditions as listed in Table 1. The BGH01exhibited broad diffraction peaks centered around 26.6° and 43.1° (2θ), which is the signature diffraction pattern of turbostratic boron nitride (t-BN). The broad peak centered at around 26.6° was originated from the (002) reflection plane, and the 43.1° broad peak corresponds to the (10) reflection plane induced by hexagonal boron nitride (h-BN) [37]. The XRD pattern of BMH was dominated by two broad diffraction patterns at 2θ of approximately 25.4° and 42.4°, which are signature patterns for the (002) and (10) bands, respectively of the hexagonal crystal structure of graphite [38]. The commonly denoted (10) band is also associated with the 2D reflection of turbostratic carbon [39]. Moreover, the absence of a peak at 2θ around 10.9° and the emergence of a broad band at around25.4° was due to the incorporation of dopants or impurities within the structure of graphene or graphite oxides [40–45]. Therefore, it is reasonable to propose that the dominant structure of BMH is doped graphene oxides.

### Structural Analysis of BGH (Guanidine Series)

**Fig. 3:**
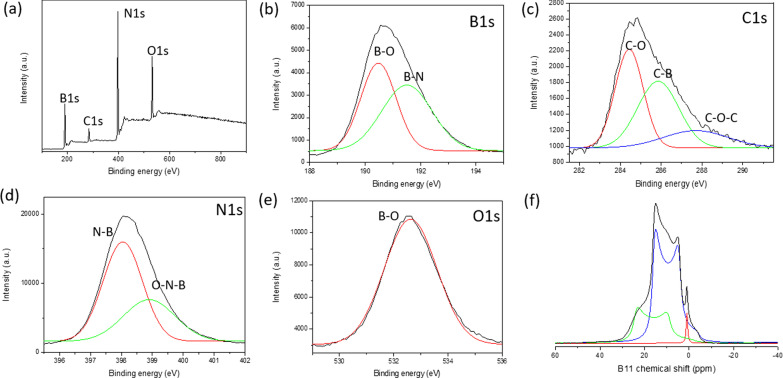
**a** XPS survey spectra of BGH01. Core level spectra of **b** B_1s_, **c** C_1s_, **d** N_1s_, and **e** O_1s_. Each deconvoluted peak is fitted with Gaussian functions. **f** The ^11^B solid-state MAS NMR spectra of BGH01. (^11^B solid-state MAS NMR of BGH01-LT is shownin Additional file 1: Figure S10)

The XPS, FTIR, and solid-state NMR spectroscopy were performed to produce deeper insight into the molecular structure of BGH. The XPS was utilized to confirm the presence of the core level electrons of the B, C, N and O elements and their respective chemical bonding in BGH compounds. Figure 3a–e show a typical XPS spectra of the BGH nanodisks. According to the XPS surface elemental composition analyses, BGH contained a high B and N content (approximately 40% each), with a lower C and O compositions of approximately 8% and 13%, respectively (Additional file 1: Table S3). The near 1:1 stoichiometry of the B and N composition is commensurate with the XRD analyses, which confirmed that BGH01 prepared in our laboratory was composed of a turbostratic boron nitride structure. All XPS spectra were fitted with a Gaussian function with *R*^2^ > 0.99, which is represented by the red and green curves in the XPS spectra for each element. For the BGH series, the B_1s_ spectra were deconvoluted into two fitted curves, which corresponded to B-O bonding (at 189.7 eV binding energy) and B-N bonding (at 190.7 eV binding energy). The N_1s_ spectra were fitted with two Gaussian curves composed of N-B bonding with 397.3 eV binding energy and B-N–O bonding with 398 eV binding energy [46]. Both N-B and B-N–O bonding indicated the presence of O-doped hBN or the formation of a BNO compound [47]. Since the synthesis of BGH was performed under atmospheric conditions, an oxygen atom was also incorporated into the h-BN domain for B-O bonding as reported in the literature [47]. This hypothesis was confirmed by analyzing the O_1s_ spectra, which showed a single peak corresponding to the B-O bonding (binding energy at 532 eV). This result further supports our initial hypothesis that the BGH series is composed of O-doped hBN, and the majority of the oxygen atom is bonded to the boron atom. The C_1s_ of the BGH spectrum was deconvoluted into three C species, namely C-O, C-B, and C–O–C, which bonded at 283.7 eV, 285 eV, and 287 eV, respectively. Due to the low composition of C and a 1:1 stoichiometry of B and N in the BGH series, we further speculated that the structure of BGH01 is C andO-doped h-BN. The proposed structure was supported by the formation of C-B, B-O, and B-N–O bonding within the hBN domain, as evidenced in the XPS spectra (Fig. 3). Thus, it is reasonable to deduce the structure of the BGH nanodisks prepared at 800 °C as C and O-doped hBN. Furthermore, powder XRD and high-resolution TEM supported the formation of covalently bonded B, C, N, and O in a honeycomb lattice with lattice spacing similar to that of turbostratic boron nitride. Throughout this manuscript, the structure of BGH01 will be referred to as BCNO-doped hBN.

We also investigated the effect of calcination temperatures and times on the structure-photocatalytic activity of BCNO nanostructures while keeping the other synthesis parameters constant. Upon increasing reaction temperature (from 600°C to 800°C) and increasing the reaction times from 30 min to 12 hrs, there was an overall increase in B-N and B-O bonding (Additional file 1: Figure S6). In contrast, B-C bonding decreases with increasing reaction temperature and time, which implied the formation of energetically stable hexagonal B-N and B-O bonds while sacrificing the metastable B-C bonds [48]. As anticipated, the B-C bonding composition increases upon increasing the ratio of hexamethylenetetramine precursor (as the C source) while keeping the other parameters constant [49]. A more detailed trend of the chemical bonding evolution of BGH prepared at different calcination temperatures, times, and precursor ratios are provided in the ESI (Additional file 1: Figure. S6 and S7).

^1^ The ^11^B solid-state MAS NMR was utilized to quantitatively analyze each B-related bonding composition in BGH and BMH quantitatively. Herein, we present the first detailed structural characterization of BCNO at the molecular level utilizing solid-state MAS ^13^C and ^11^B NMR to gather specific C- and B-related bonding. Although hBN nanomaterials and their defect-induced electronic properties have attracted significant interest, the molecular structure of hBN edges and defects structure are largely unknown [50]. The lack of structural characterization of boron-related nanomaterials is due to the difficulty in analyzing the solid-state ^11^B NMR spectrum because ^11^B is a half-integer quadrupole nucleus (*I* = 3/2) [51, 52]. Solid-state ^11^B NMR is also challenging to interpret due to the second-order quadrupole coupling resulting in the signal's distortion, which can only be partially averaged via MAS NMR [53]. Furthermore, the range of chemical shift for ^11^B NMR is relatively narrow, making peak assignment of the broad, overlapping, and distorted peaks of various boron species extremely challenging [54]. In this study, we performed ^11^B solid-state NMR recorded at 9.4 T, and the spectrum was deconvoluted using topspin solid-line shaped analysis (SOLA). By following the CP-MAS ^11^B NMR experiment reported in the literature, we obtained meaningful B-related chemical bonding information by taking into account the quadrupolar coupling constant (*C*_Q_) and the electric field gradient (EFG) tensor asymmetry (*η*_Q_) [51, 52, 55]. Figure 3f shows the solid-state ^11^B spin-echo NMR spectra of BGH with three major peaks and *δ*_iso_ centered at 28.3 ppm, 20 ppm, and 1.2 ppm, respectively. Based on literature studies on CP-MAS ^11^B NMR of boron nitride and their related structures, the peak with a *δ*_iso_ of 28.3 ppm (green trace fit) and a *C*_Q_ of 2.85 MHz corresponded to the trigonal-planar BN_2_(OH) species with a single hydroxyl group [53–55]. When  the hydroxyl group or oxygen bridging atom replaced the nitrogen atom around the trigonal-planar boron sites, a new B species with a *δ*_iso_ of 20 ppm (blue trace fit) appeared. This lower chemical shift signal was most likely attributed to another trigonal boron site with two hydroxyl groups or one hydroxyl group and one bridging oxygen atom (BN(OH)_2_ or BNO(OH) sites). The sharp peak at *δ*_iso_ of 1.2 ppm (red trace fit) corresponds to a four-coordinate tetrahedral B site, likely coordinated by nitrogen and multiple hydroxyl groups or a bridging oxygen atom (52–54) or carbon-related C-B bonding [46, 52]. The CP-MAS ^11^B NMR analysis of BGH01 revealed various B-N, O-B, and B-C bondings commensurate with the XPS and XRD analyses. Furthermore, solid-state ^11^B NMR also revealed that most boron was bonded with nitrogen and with one or more hydroxyl groups as the BN_*x*_(OH)_3−*x*_ species. These hydroxylated species were speculated to provide colloidal stability in the aqueous solution through hydrogen bonding and electrostatic stabilization. We also observed a reduction in the tetracoordinate B-species such as BN_*x*_(OH)_4−*x*_ or BN_*x*_(O)(OH)_3−*x*_ (in which O is the bridging oxygen) and the boroxol rings [54, 56] with increasing reaction temperature. Concurrently, various tricoordinate BN_*x*_(OH)_3−*x*_ species emerged when the  annealing temperature increased from 600°C to 800°C (Additional file 1: Figure S8 and S10). This result implied that at high temperatures, the boroxol ring reacted with ammonia to form various hydroxylated BN_*x*_(OH)_3−*x*_ or BN_*x*_(O)(OH)_2−*x*_ species [55] (Additional file 1: Fig. S10).

### Structural Analysis of BMH

**Fig. 4:**
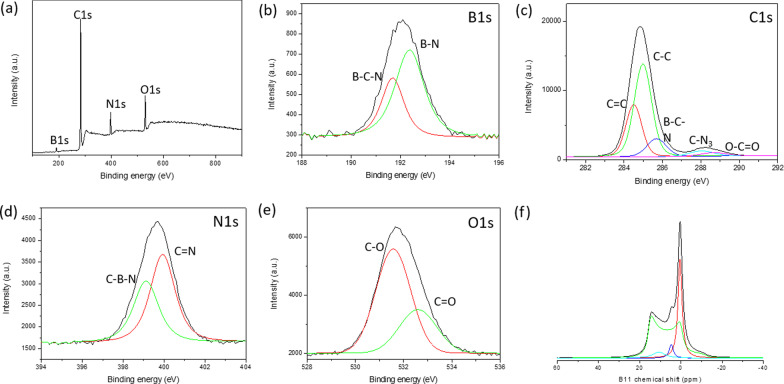
**a** Survey XPS spectra of BMH01 and ^11^B NMR spectra. Core level spectra of **b** B_1s_, **c** C_1s_, **d** N_1s_, and **e** O_1s_. Each core spectra were fitted with a black trace, while the red and green traces under the peak were deconvoluted using a Gaussian function. **f** ^11^B solid-state MAS NMR were deconvoluted using SOLA analysis to tricoordinate and tetracoordinate B-sites. The XPS and ^11^B solid-state MAS NMR of other BMH series compounds can be found in Additional file 1: Figure S7, S11, and S12)

**Fig. 5:**
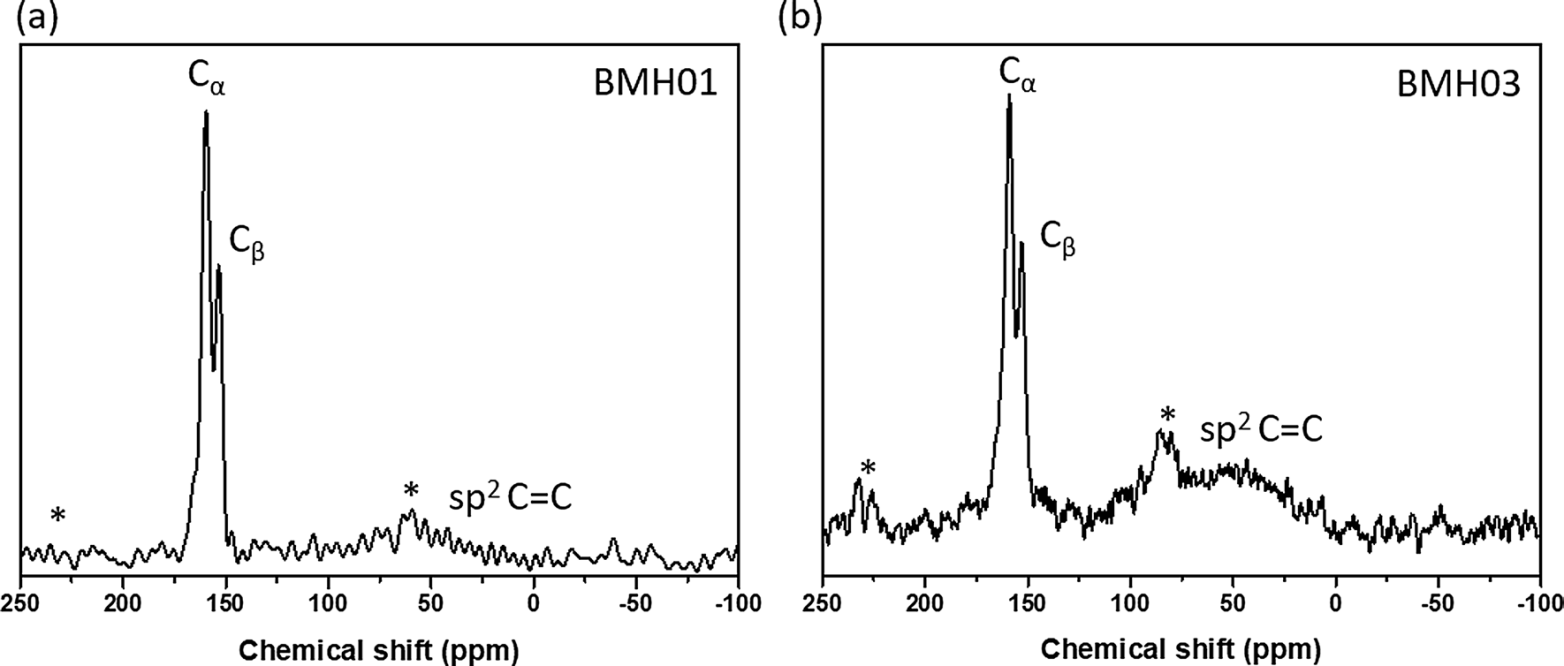
Solid-state ^13^C MAS NMR of **a** BMH01 and **b** BMH03

Based on our vigorous structural analysis via XPS and FTIR as well as ^11^B, and ^13^C solid-state MAS NMR, the structure of BMH was proposed to be BCNO-doped graphene oxides. According to the XPS surface elemental analysis, BMH was composed of to 75% of carbon species and only 5% of boron-related species (Additional file 1: Table S3). The deconvoluted B_1s_ spectra showed BCN and BN bonding at 191.7 and 192.4 eV binding energies, respectively (Fig. 4). The C_1s_ species showed two distinct photoemission signals, which corresponded to C–C bonding (sp^2^ and sp^3^ C–C bonding) with binding energy at approximately 285.0 eV, and a weaker component arose from the B-C-N bonding appearing at 285.6 eV [57]. The other relatively small signals at 288.0 eV and 288.7 eV were due to C-N_3_ and C = O bonding, respectively [46, 57, 58]. The oxygenated C atom (C = O) was speculated to form at the edges of the graphene oxide domains, and the C-N_3_ bonding was the characteristic peak for CN. The N_1s_ XPS spectra, centered at 399.0 eV binding energy, can be fitted with two Gaussian curves composed of C-N-B at 399.2 eV binding and C = N bonding at 400.0 eV [46, 57, 59, 60]. Based on XPS analysis, the BMH series comprised only about 10–25% of O-related bonding compared to approximately 35% of O-related bonding in the BGH series. The O_1s_ spectra centered at 531.2 eV binding energy can be deconvoluted into C = O and CO bonding, which could be attributed to the graphene oxides domain. The ^13^C solid-state CP-MAS NMR further showed the presence of graphitic C = C bonding at 40.2 ppm, which corroborates with the XRD and XPS results [38] (Fig. 5). In each of the BMH series reported in this paper, the ^13^C NMR showed two equimolar ratios of carbon species at 160 and 154 ppm corresponding to *C*_α_ and *C*_β_, respectively, which is typically found in graphitic carbon nitride structures [61] (Fig. 5 and Additional file 1: Table S4). Compared to the bulk CN synthesized according to the literature procedure [34], the chemical shift of the signature resonance for *C*_α_ and *C*_β_ peak appeared at 164 and 156 ppm, respectively (Fig. S13). Boron doping into the CN heptazine structure could have contributed to these slight chemical shift differences between the BMH series and the bulk CN (Additional file 1: Fig. S13).

Due to the abundant  evidence of the presence of carbon nitride (CN) in the BMH series based on solid-state NMR spectroscopy (Fig. 5), we considered three possibilities of interactions between BCNO-doped graphene oxide and CN in BMH, namely: (i) bulk phase separation, (ii) disordered 2D network, and (iii) layered intercalation [62]. To eliminate the possibility of bulk phase separation, we examined the XRD pattern of CN nitride andBMH as well as a physical mixture of both in a 1:1 mass ratio. We found that the XRD patterns of the mixtures showed only the diffraction pattern of the bulk CN with a slight reduction in the crystallinity compared to the pristine CN diffraction [63] (Additional file 1: Fig S4). Since the XRD pattern of BMH did not possess any diffraction peaks that corresponded to CN, this experimental result confirmed that CN did not form as a bulk-separated domain during the synthesis of BMH (Additional file 1: Fig. S4). We also considered the formation of a disordered 2D network, in which CN and the doped-graphene oxides are bonded on the same 2D plane [62]. Based on a literature report on a CN/graphene oxide 2D matrix, the XRD pattern of a disordered 2D network showed characteristic peaks for both species with a slight peak broadening and a slight peak shifting [64]. However, the XRD pattern of BMH (Fig. 2) did not contain any signature diffractions of CN. A previous study also showed that characteristic peaks of graphene oxide disappeared in a graphitic CN/amorphous CN/graphene oxide composite due to the layer-by-layer interactions [65]. Therefore, it is reasonable to propose that CN is intercalated between the doped-graphene oxide layers. The FTIR spectrum of the selected BGH and BMH series is shown in Additional file 1: Fig. S5.

Boron-related bonding within the BMH series was investigated using ^11^B solid-state MAS NMR at 9.4T, and the broad NMR spectrum was deconvoluted using the SOLA analysis showing the presence of both tricoordinate and tetracoordinate boron site. The SOLA analysis yielded four line fittings under the broad ^11^B NMR spectrum, which could be assigned as trigonal planar BN_2_(OH) or BN_2_O at a δ_iso_ of 19.8 ppm and a CQ of 2.85 MHz (green fitting). The bay and corner B sites as in B-doped CN appeared at a *δ*_iso_ of 5 ppm and *δ*_*iso*_ of 11 ppm, respectively [61]. These assignments are also commensurate with the formation of CN based on the ^13^C NMR and XPS analyses. Compared to the BGH series, the relative composition of the tetracoordinate B(IV) site of BMH was much higher (ca. 55% in the BMH series vs. 3% in the BGH series). However, the tetracoordinate B(IV) species in BMH appeared at a lower chemical shift than those found in BGH (Fig. 3f) and was therefore presumed to be the BN_2_(OC)_2−*x*_(OH)_*x*_ species [46]. Notably, the h-BN domain was absent from the BMH series prepared via thermal annealing at a lower temperature (600 °C). However, upon increasing the thermal annealing temperature from 600 °C to 800 °C, the structures of BMH01HT-30 min and BMH01HT-12 h showed a high composition of tetracoordinate BN_2_(OH)_2_ species and the tricoordinate BN_3_ bonding (Additional file 1: Fig. S11 and S12). The presence of a high composition of tetracoordinate BN_2_(OH)_2_ and BN_3_ bonding was shared among all the inactive BCNO investigated in this study. Moreover, although BMH01HT-12 h possessed an identical surface elemental composition to that of BGH01, the solid-state ^11^B NMR revealed that both compounds possessed significant structural differences, which explained for their differences in photocatalytic activity (Additional file 1: Table S3 and Fig. S12).

In light of the moderate photocatalytic activity of BMH01 and BGH01 (Fig. 7), further synthesis optimization was performed to expand their light absorption spectrum into the visible light region. Previous literature showed that increasing the composition of the hexamethylenetetramine precursor (as a carbon source) could modulate the bandgap and photoluminescence properties of BCNO. Based on these reports, BMH and BGH compounds with a higher ratio of hexamethylenetetramine were prepared accordingly while keeping the other parameters constant. The optimized BCNO with a higher carbon content is denoted as BMH03 and BGH03, in which the molar ratio of carbon source was increased from 0.1 to 0.3. The higher ratio of hexamethylenetetramine precursor yielded BMH03 with a higher composition of the graphitic domain as in sp^2^ C = C, and a small peak emerged which corresponded to BCN bonding at 191 eV binding energy (Additional file 1: Fig. S7). The increase in the graphitic sp^2^ C = C domain upon increasing the concentration of hexamethylenetetramine is consistent with the role of hexamethylenetetramine as both a C and N source in the synthesis of N-doped graphite [66]. The increased sp^2^ C = C graphite bonding in BMH03 was further confirmed via ^13^C CP-MAS NMR with the emergence of a more prominent peak centered at 40.2 ppm, which is the signature of graphitic C = C(H) bonding (Additional file 1: Table S4).

### Optical Properties

**Fig. 6:**
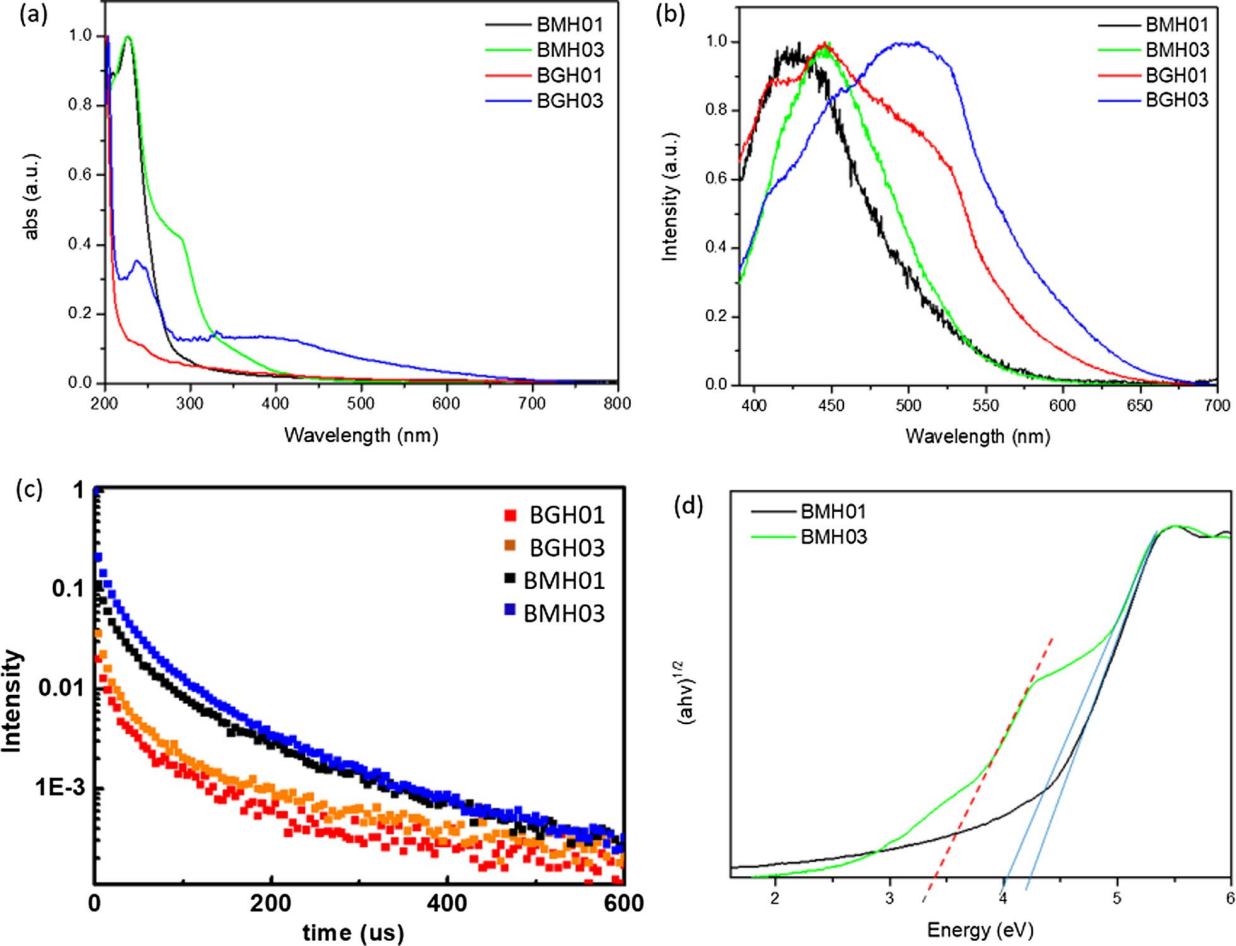
**a** Overlay UV absorbance spectra of BMH01, BMH03, BGH01, and BGH03. **b** Stacked photoluminescence spectra of BMH01, BMH03, BGH01, and BGH03 upon excitation at 365 nm **c** stacked photoluminescence lifetime decay spectrum of BMH01, BMH03, BGH01, and BGH03 monitored at a predetermined *λ*_max_ for each sample. Samples were excited with 337 nm laser pulses at 298 K, **d** Overlay Tauc plot (αhv)^1/2^ vs. h*v,* for BMH01 (black trace) and BMH03 (green trace). The optical band gap is represented by  thesolid line, while the interband state for BMH03 is represented by the red  dashed line

The optical properties of BMH and BGH were investigated using UV–visible absorption and photoluminescence spectroscopy, as shown in Fig. 6. Since BGH and BMH series possessed distinctively different structures, and the optical properties of nanomaterials are highly correlated with their structures, the origin of absorption and luminescence for both series were also found to be different. In this study, BGH01 quasi-spherical nanoparticles showed a featureless UV–vis spectrum. (Fig. 6a, red trace). As the ratio of hexamethylenetetramine increased, the intensity of absorbance peaked at 237 nm for BGH03 and increased with the emergence of an additional broad absorption peak centered around 330 nm (Fig. 6a, blue trace). The origin of the optical properties of BCNO is controversial due to the lack of structural analysis of BCNO nanomaterials. The most widely cited origin of photoluminescence of BCNO is attributed to the formation of B, C, N, and O self-interstitial sites, substitutional impurities, and native point defects within hBN or the graphene matrix [50, 67, 68]. Other possible photoluminescence mechanisms in the BCNO system include the electronic transition from the nitrogen-vacancy (V_N_) levels to the carbon impurity levels, the electronic transition between the closed-shell BO^−^ and BO^2−^ anions, and intrinsic state emission and defect state emission (surface energy traps). The featureless UV absorbance of BGH01 can be attributed to the electronic transition between the valence band (VB) and conduction band (CB) of boron nitride with a bandgap energy of ca. 5.9 eV. The lower energy absorption in BGH03 was a result of the mid-gap absorption from the valence band to the nitrogen-vacancy (V_N_) level located approximately 0.7–1.0 eV below the conduction band of hBN.(62) The absorption peak at ca. 330 nm could be attributed to the presence of C-related impurities level located ca. 2–4 eV below the conduction band of hBN [69–71]. Under 365 nm excitation, BGH01 produced a broad emission with three bumps located at 412 nm, 445 nm, and 489 nm, respectively. Based on the BGH structural analysis, in which the dominant structure was composed of BN and BO-related bonding, the photoluminescence of the BGH series could be most likely originated from the B-O luminescence centers [24, 47, 72]. The yellow-green emission at 445 and 489 nm could be induced by the transition from the V_N_ level to the carbon-related and oxygen defect levels (2–4 eV) below the conduction band of h-BN [69–71]. As the C composition increased in BGH03, the emission wavelength was further red-shifted to 506 nm, consistent with literature reports [73, 74]. The stacked photoluminescence (PL), UV absorbance, and Tauc plot for different BGH series prepared in this study are presented in Additional file 1: Fig. S14.

Based on XRD and various spectroscopic analyses, the structure of BMH was deduced to be dominated by BCNO-doped graphene oxides (Fig. 4, Additional file 1: Fig. S7, and Fig. S12, and Table S4). Thus, the optical properties of the BMH series are hypothesized to be more closely related to the carbon-quantum dot (CD) [75, 76] and doped-graphene oxides systems [44, 77–79]. Based on the origin of photoluminescence of the CD and graphene oxides, the optical properties of BMH prepared in this study can be attributed to the intrinsic state emission [76, 80, 81], electron–hole recombination [82, 83], and defective state emission [84]. Intrinsic emission of BMH is speculated to have originated from isolated sp^2^ luminescence centers embedded within the sp^3^ matrix of the carbonaceous film. The sp^3^ matrix of graphene oxides is composed of C–OH, C–O–C, and C = O edge sites, whose energy levels lie between the energy levels of π–π* states of the sp^2^ C = C domain, thus giving rise to multiple absorption bands. Both BMH01 and BMH03 possessed a strong UV absorbance band at ca. 240 nm, corresponding to the π to π* transition of C = C within the graphene oxides domain. With the increasing ratio of hexamethylenetetramine in the BMH03 sample, an additional bump at 288 nm emerged, which can be ascribed to the n-π* transition of the C = O and C = N bonds of the oxidized graphitic region [77, 85, 86]. The latter absorbance band at ca. 288 nm was induced by oxygen, nitrogen, and boron defect sites, creating new radiative recombination sites [82, 87–89]. Upon photoexcitation at 365 nm, BMH01 and BMH03 exhibited a maximum emission wavelengths at 429 and 447 nm, respectively. The BMH03 sample showed a slight red-shift emission, unlike BMH01 due to the greater extent of graphitization [77, 84] (Additional file 1: Figs. 5 and 6). Both BMH samples revealed a broad and much lower energy emission wavelengths than BGH samples due to lower energy emissive centers arising from O, N, and B defects and surface states. According to the electron–hole recombination mechanism, these photoexcited electrons from each defect state recombine with their corresponding holes in the HOMO, thus yielding a broad photoluminescence emission [90] (Fig. 6b). Interestingly, only BMH01 exhibited a pronounced excitation-dependent photoluminescence as observed in other BCNO [72] and carbon quantum dot systems [76]. The presence of O, and N impurities embedded within the graphene oxides matrix was shown to create a large number of surface emissive traps that corresponded to a diverse energy levels within the bandgap, thus yielding an excitation dependent fluorescence spectra in BMH01 (Additional file 1: Fig. S15). In contrast, the lack of an excitation dependent emission in BMH03 could be explained by the formation of a greater extent of graphitization (C = C) with a concurrent reduction in the surface states population (eg.: C = O) [44].

The bandgap values of BMH and BGH prepared in this study were estimated from Tauc's formulation: (αhν)^2^ − hν, where α is the absorbance (Fig. 6d). The bandgap was estimated by extrapolating the photon energy intercept at (αhν)^2^ = 0. For the BMH series, the presence of multiple energy levels within the optical bandgap may have originated from the electronic transition from various π-π* (C = C bonds) and n-π* of C = O or other surface groups [76, 82, 87]. As for the BGH series, carbon substituted on boron sites (C_B_), nitrogen-vacancy sites (V_N_), and interstitial carbon defect levels gave rise to the emergence of interband states between the bandgap of hBN. The presence of multiple energy levels was supported by the photoluminescence spectra of lower energy radiative recombinations [24, 47, 66] (Fig. 6b and Table 2). The BGH03 sample exhibited two large bandgaps at 5.7 eV and 3.8 eV, corresponding to the bandgap of hBN and the transition from the valence band to V_N_ levels, respectively [50, 74] (Additional file 1: Fig. S14).

**Table 2:** Optical bandgap energy and interband states estimated based on Tauc's formulation

BCNO	Optical band gap (eV)	Interband state (eV)
BMH01	4.2	–
BMH03	4.0	3.3
BGH01	5.7	–
BGH03	5.7	3.8

Tauc plots of BGH01 and BGH03 are available in Additional file 1: Fig. S14

Long-lived charge carriers that can persist into the microseconds and milliseconds timescales in semiconductor photoelectrodes such as CN photocatalysts, have been proposed as an important parameter in enhancing photocatalytic activity by reducing charge recombinations [91–95]. Time-resolved photoluminescence (TRPL) experiments were conducted to gain insight into the recombination processes of the photogenerated charge carriers of BCNO (Fig. 6c). The µs-PL decay kinetics could be fitted with three exponential decays according to the following equation.

$$I\left( t \right) = I_{1} \exp ( - t/\tau_{1} ) + I_{2} \exp ( - t/\tau_{2} ) + I_{3} \exp \left( {t/\tau_{3} } \right)$$

The multiple exponential decays imply that BCNO undergoes complex recombination from both intrinsic and defect states of BCNO [32, 72]. In the equation,  *I*_1_ through *I*_3_ are constants with values of emission intensity measured at *t* =0, and*τ*_1_ through *τ*_3_ are the lifetimes of three channels responsible for the decay, respectively. Through multiexponential fitting of the entire decay curves for the BMH and BGH series, the average lifetimes were calculated to be 1.58, 2.10, 5.18, and 8.14 µs for BGH01, BGH03, BMH01, and BMH03, respectively. The values of *I* and *τ* in Eq. 1 for the BMH and BGH series are reported in Additional file 1: Table S5. The persistent lifetime of the charge carrier in the BCNO system has been attributed to the presence of shallow traps composed of nitrogen-vacancy (V_N_) stabilized by carbon impurities, which were located ca. 0.7 eV-1.0 eV below the conduction band of h-BN [72, 74]. Shallow traps in CN photocatalysts have been attributed to charge separation states with long life-times due to chemical defects [95]. According to works related to prolonged photoluminescence in CN and other nanostructured photoelectrodes [91, 92], the microseconds lifetimes of BCNO-doped graphene oxides and BCNO-doped hBN are associated with the enhanced charged separation within the BCNO domain. The ultralong lifetimes are speculated to be a critical factor in facilitating heterogeneous photocatalysis [91].

### Photocatalytic Dye Degradation

**Fig. 7:**
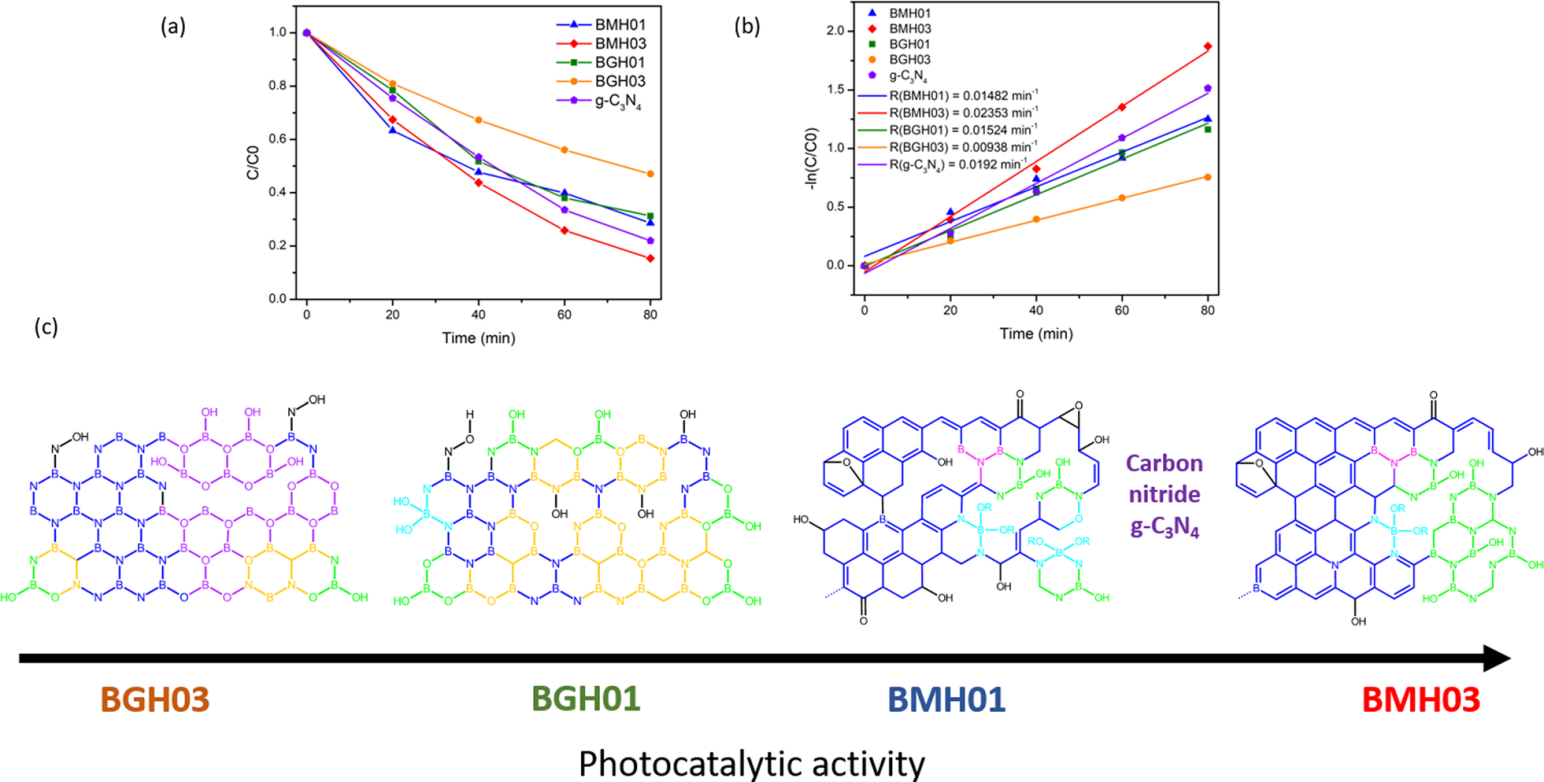
UV–visible light-induced photocatalytic MB degradation using BGH and BMH. **a** Photodegradation of MB under UV–visible light (plot of *C*/*C*_0_) and **b** pseudo-first-order rate reaction kinetics for MB dye using BGH, BMH, and CN as a photocatalyst. **c** The proposed structures for BGH01, BGH03, BMH01, and BMH03 and their corresponding activities toward photodegradation of MB dye. Each domain is color-coded, i.e., BCNO (orange), h-BN and graphene oxides (blue), tricoordinate boron BN_2_(OH) and BO_2_(OH) (green), boroxol ring (purple), tetracoordinate B sites (aqua blue), and functional groups or dangling bonds (black).

As a proof of concept demonstration, the photocatalytic performance of BMH and BGH was evaluated by the photodegradation of methylene blue (MB) under UV–visible light irradiation. Details of the experimental procedure and analysis of the photocatalytic dye degradation are available in the ESI. Figure 7 shows the percent degradation (*C*/*C*_0_ × 100%) for BMH03 (red line), BMH01 (blue line), BGH01 (green line), BGH03 (orange line), and CN (purple line), where C is the concentration of MB at a time, *t* and *C*_0_ is the initial concentration MB after the dark equilibrium. According to the Langmuir–Hinshelwood model, ln(*C*/*C*_0_) = *kt*, where *k* is the rate constant, the dye degradation rate constant values were calculated to be 2.31 × 10^−3^ min^−1^ for BMH03, 1.52 × 10^−3^ min^−1^ for BGH01, 1.48 × 10^−3^ min^−1^ for BMH01 and 9.38 × 10^−4^ min^−1^ for BGH03. Compared to the state-of-the-art metal-free photocatalyst, BMH03 exhibited a 25% improvement in the photocatalytic dye degradation rates (Fig. 7). This proof-of-concept demonstration warrants a more in-depth investigation of the structure–property relationship of this new metal-free, boron-based photocatalyst.

**Fig. 8:**
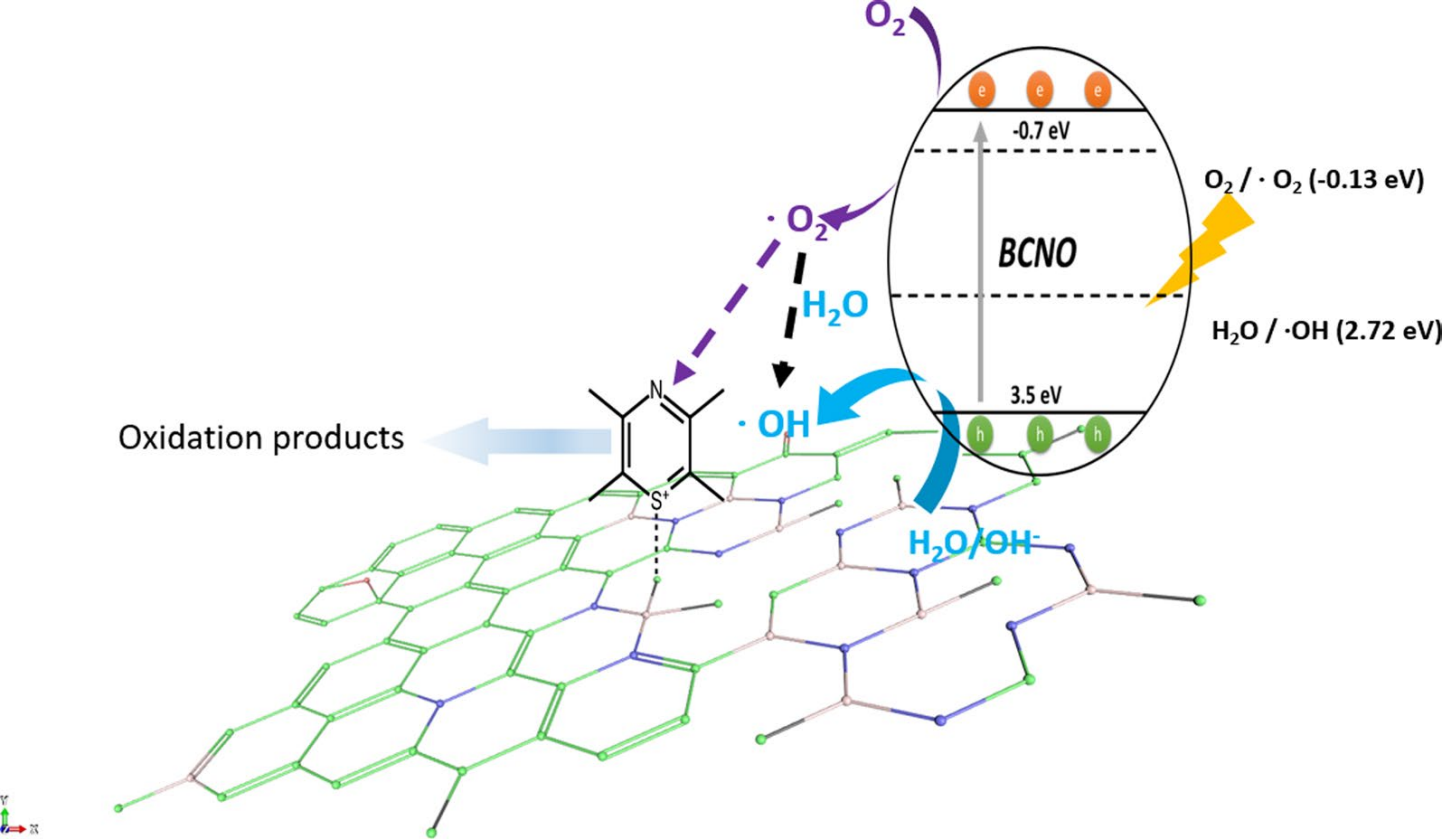
Schematic illustration of the proposed mechanism of BCNO in catalyzing the degradation of MB upon light irradiation. The positively charged MB was selectively absorbed on the N_2_B-OH sites. The band structures of BMH03 were determined via UPS and Tauc plot (Fig. 6 and Additional file 1: Fig. S17). The redox potentials of O_2_/O_2_ = − 0.13 eV vs E_NHE_ and H_2_O/OH = 2.72 eV vs. E_NHE_ are given in black dashed lines as reference

Based on the detailed structural analysis and the proposed structure in Fig. 7c, the highest photocatalytic activity of BMH03 consisted of BCNO-doped graphene oxides [1, 65]. This ternary metal-free photocatalyst is reported to enhance the photocatalytic performances by increasing the charge separation and migration to the reaction site [1]. Additionally, the incorporation of boron into graphene-based materials [11, 20], CN [21, 96], and carbon nanotubes [97] has also exhibited enhanced performance compared to their pristine material without a dopant due to multiple synergistic effects. The large differences in electronegativity between boron, carbon, and nitrogen (2.04 vs. 2.55 vs. 3.04, respectively) yielded a strongly polarized bonding towards C and N atoms. As a result, a local positive charge was formed on boron that turned boron into a strong acidic defect site for preferential adsorption sites of pollutants [98], O_2_ [97], HOO^−^ and OH^−^ [99, 100]. Therefore, electron-rich pollutants such as MB (used as a model reaction) were speculated to preferentially adsorbed onto the electropositive B sites of the BMH photocatalyst. The photocatalytic degradation of MB on BCNO was proposed to undergo an indirect dye degradation mechanism [101]. In the indirect photodegradation mechanism, the photogenerated holes on the surface of BCNO produced highly oxidative hydroxyl radicals and attack the  bond of MB. Meanwhile, photogenerated electrons from the conduction band of BCNO formed highly reducing superoxide radical anion O_2_^−^ species that could directly attack MB. Figure 8 illustrates the proposed mechanism of BCNO in photocatalyzing the degradation of MB

According to the XPS and solid-state NMR analyses, BMH03 possessed a higher composition of graphitic sp^2^ C = C with a simultaneous reduction in tetracoordinate B site compared to BMH01 (Additional file 1: Fig. S9 and Table S4) The high composition of tetracoordinate B-sites in BMH01 (33%) also translated to a reduction in the number of boron Lewis acid sites serving as the active catalytic center, which explains the trend of photocatalytic activities among the BMH series [13]. However, the BGH series comprised the C, and O-doped h-BN domain with various tri- and tetracoordinate boron sites. Interestingly, BGH01 annealed at a lower temperature (Table 1, BGH01LT) was found to be inactive but exhibited a high absorbing ability in removing dye from the solution (Additional file 1: Fig. S1 for TEM morphology; Fig. S16 for photodegradation MB UV–vis absorbance).

The previous report also concluded that BCNO was photocatalytically inactive towards dye degradation but exhibited an excellent absorbing ability in removing dye from the solution [102]. The inactive BGH01LT was comprised primarily of tetracoordinate B sites and boron oxides-related bonding. Based on  the SOLA analysis and the literature , the tricoordinate boron site with a *δ*_iso_ of 16.7 ppm was related to boroxol rings (B_2_O_3_) [54, 56]. Our results are also supported by other works on the catalytic activation of peroxymonosulfate using amorphous boron [22]. However, the photocatalytic active BGH01, BMH01, and BMH03 possessed a high composition of BN(OH)_2_ and BN_2_OH bonding sites. Our investigation suggested that the photocatalytic activity of BCNO is highly dependent on the local structure of the boron site. While the exact catalytic site and mechanism are yet to be explored, we proposed that BN_*x*_(OH)_3−*x*_ served as one of the BGH and BMH series catalytic sites. For the BMH series, the formation of tetracoordinate B-O sites was detrimental in catalyzing dye degradation due to the reduction in the Lewis acid site. At the same time, the increasing composition of the sp^2^ C = C graphitic domain enhanced the photocatalytic activities of the BGH and BMH series.

## Conclusions

In summary, BCNO structures and their photocatalytic activities have been presented here and found to be highly dependent on the choice of precursor, precursor ratios, annealing temperatures, and times. In this study, two types of distinctly different BCNO nanostructures were prepared via low-temperature annealing (600 °C–800 °C): (1) BCNO-doped boron nitride and (2) BCNO-doped graphene oxides. Through systematic investigation of using two different nitrogen precursors, crystalline BCNO with a quasi-spherical shape was prepared at 800 °C for 12 hr using guanidine hydrochloride as the nitrogen source. This series of BCNO exhibited moderate photocatalytic activity through the emergence of BN_2_(OH) or BN(OH)_2_ tricoordinate boron serving as the Lewis acidic site. However, BCNO prepared using melamine as the nitrogen source at 600 °C yielded multi-layered sheets with ill-defined shapes. These BCNO-doped graphene oxides layered structures exhibited the highest photocatalytic activity, surpassing the state-of-art metal-free photocatalyst, CN. For the melamine-derived BCNO layered structures, the presence of tricoordinate boron species as BN_2_(OH) or BN(OH)_2_ and a higher composition of graphitic sp^2^ C = C were speculated to play an important role in promoting their photocatalytic activity. This study demonstrates the potential of BCNO as a photocatalyst for energy conversion and environmental remediation applications. Further structural optimization on this new B-C-N–O photocatalyst system is expected to facilitate the development of a sustainable catalyst for applications including solar hydrogen fuel production, environmental remediation, electrocatalytic oxygen reduction reaction, and catalytic oxidative dehydrogenation reaction.

## Supplementary Information

**Additional file 1.** **Table S1:** Different sets of precursors used in the preparation of BCNO. **Table S2:** List of experiment and BCNO sample names investigated in this study. **Figure S1:** TEM images of (a) BGH01LT, (b)BGH01-30min, (c) BGH01-6hr, and (d) BGH02 in low magnification, (e) BGH02 in high magnification, (f) BGH03 in low magnification, (g) BGH03 in high magnification. (h) EDS mapping analysis of BGH03 (1) B (2) C (3) N (4) O. (i) BGH01LT, (j) BGH01-30min, (k) BGH01, and (l) BGH03 under UV lamp (365 nm, 4 watts). **Figure S2:** TEM images of (a) BMH01-30min, (b)BMH01HT, (c) BMH02. (d) EDS mapping analysis of BMH03. (1) B (2) C (3) N (4) O. (e) BMH01-30min (f) BMH01HT (g) BMH01 and (h) BMH03 under UV lamp (365 nm, 4 watts). **Figure S3:** Stacked XRD of (i) BGH01LT, (ii) BGH02, (iii) BGH03, (iv) BMH01-30min, (v) BMH01HT, (vi) BMH02. **Figure S4:** (a) Stacked XRD (normalized intensity) of g-C3N4, BMH01, and the physical mixture of both. (b) Stacked XRD (normalized intensity) of g-C3N4, BGH01, and the physical mixture of both. **Table S3:** XPS surface elemental composition BGH01, BGH03 and BMH01, BMH03. **Figure S5:** (a) Stacked FTIR of BGH01 (blue trace), BGH03 (red trace) and BGH01LT (green trace) and their corresponding IR active functional groups. (b) Stacked FTIR of BMH01 (blue trace), BMH03 (pink trace) and BMH01-30min (orange trace) and their corresponding IR active functional groups. **Figure S6:** Evolution of bonding composition, B-N, B-C and B-O in BGH series with reaction temperature and time based on XPS analyses. **Figure S7:** (a) Survey XPS spectra of BMH03, and 11B NMR spectroscopies. Core level spectra of (b) B 1s, (c) N 1s, (d) C 1s, (e) O 1s. Each core spectra were fitted with a black trace, while the red and green traces under the peak were deconvoluted using a Gaussian function. (f) 11B solid state MAS NMR were further deconvoluted with topspin SOLA software. **Figure S9:** Evolution of tetracoordinate boron site for BMH series based on 11B solid state MAS NMR analysis. **Figure S8:** Evolution of tetracoordinate boron site for BGH series based on 11B solid state MAS NMR analysis. **Figure S10:** Solid-state 11B NMR for BGH01LT showing a high composition of tetracoordinated BN2(OH)2 bonding, along with the high composition of boron oxides. **Figure S12:** Solid-state B-11 NMR for BMH01HT showing emergence of BN3 and its corresponding oxides. The tetracoordinate boron species here changed from BN2(OH)(CO) or BN2(CO)2 to BN2(OH)2, which resembles the tetracoordinated B-sites in BGH series. **Figure S11:** Solid-state 11B NMR for BMH01-30min showing emergence of BN3 and its corresponding oxides. The tetracoordinate boron species here changed from BN2(OH)(CO) or BN2(CO)2 to BN2(OH)2, which resembles the tetracoordinated B-sites in BGH series. **Figure S13:** Solid state 13C MAS NMR of g-C3N4. **Table S4:** Carbon composition of BMH01 and BMH03 based on CP-MAS 13C NMR deconvolution data and their corresponding integration values under each peak. **Figure S14:** Overlay emission spectra of BCNO prepared in this study. (a) Stacked normalized PL spectra and (b) Stacked normalized UV-visible absorbance spectra BMH and BGH series prepared in this study. Excitation wavelength was set at 365 nm. (c) Overlay Tauc plot (αhv)1/2 versus hv for BMH01 (red trace) and BGH03 (blue trace). **Figure S15:** Photoluminescence emission of BMH01 and BMH03 at different excitation wavelength. The highest emission intensity is excited with 320 nm light for BMH01 and 340 nm for BMH03. (a) excitation dependent PL spectrum of BMH01 (b) normalized excitation dependent PL spectrum for BMH01 (c) PLE spectrum of BMH01 at 420 nm emission (d) excitation dependent PL spectrum of BMH03 (e) normalized excitation dependent PL spectrum for BMH03 (f) PLE spectrum of BMH03 at 440 nm emission. **Table S5:** Quantum yield and multiexponential decay fitting results of the µs-time resolved photoluminescence spectra monitored at λmax of each sample. Excitation wavelength was set at 337 nm **Figure S17:** UPS spectra of (a) BMH01, (b) BMH02, and (c) BMH03. **Figure S16:** UV-visible absorbance of methylene blue during dye photodegradation using BMH01-30min. **Figure S17:** UPS spectra of (a) BMH01, (b) BMH02, and (c) BMH03. **Table S6:** Energy band position of BMH series.

## Data Availability

All data are fully available without restriction.

## Abbreviations

BCNO: Boron carbon oxynitride
CN: Carbon nitride
hBN: Hexagonal boron nitride
TRPL: Time-resolved photoluminescence
MAS: Magic angle spin
CP: Cross polarization
SOLA: Solid line shape analysis
*I*: Intensity
ATR: Attenuated total reflectance

## References

[1] Rahman MZ, Kibria MG, Mullins CB (2020). Metal-free photocatalysts for hydrogen evolution. Chem Soc Rev.

[2] Hutton GAM, Martindale BC, Reisner E (2017). Carbon dots as photosensitisers for solar-driven catalysis. Chem Soc Rev.

[3] Phang SJ, Tan L-L (2019). Recent advances in carbon quantum dot (CQD)-based two dimensional materials for photocatalytic applications. Catal Sci Technol.

[4] Ong W-J, Tan L-L, Ng YH, Yong S-T, Chai S-P (2016). Graphitic carbon nitride (g-C3N4)-based photocatalysts for artificial photosynthesis and environmental remediation: Are we a step closer to achieving sustainability?. Chem Rev.

[5] Dai L, Xue Y, Qu L, Choi H-J, Baek J-B (2015). Metal-free catalysts for oxygen reduction reaction. Chem Rev.

[6] Ma R, Lin G, Zhou Y, Liu Q, Zhang T, Shan G, Yang M, Wang J (2019). A review of oxygen reduction mechanisms for metal-free carbon-based electrocatalysts. npj Comput Mater.

[7] Navalon S, Dhakshinamoorthy A, Alvaro M, Antonietti M, García H (2017). Active sites on graphene-based materials as metal-free catalysts. Chem Soc Rev.

[8] Han Q, Wang B, Gao J, Qu L (2016). Graphitic carbon nitride/nitrogen-rich carbon nanofibers: highly efficient photocatalytic hydrogen evolution without cocatalysts. Angew Chem.

[9] Safaei J, Mohamed NA, Mohamad Noh MF, Soh MF, Ludin NA, Ibrahim MA, Isahak WN, MatTeridi MA (2018). Graphitic carbon nitride (g-C_3_N_4_) electrodes for energy conversion and storage: a review on photoelectrochemical water splitting, solar cells and supercapacitors. J Mater Chem A.

[10] Wang R, Lu K-Q, Tang Z-R, Xu Y-J (2017). Recent progress in carbon quantum dots: synthesis, properties and applications in photocatalysis. J Mater Chem A.

[11] Zheng Y, Jiao Y, Ge L, Jaroniec M, Qiao SZ (2013). Two-step boron and nitrogen doping in graphene for enhanced synergistic catalysis. Angew Chem Int Ed.

[12] Liu J, Wen S, Hou Y, Zuo F, Beran GJO, Feng P (2013). Boron carbides as efficient, metal-free, visible-light-responsive photocatalysts. Angew Chem Int Ed.

[13] Fang Y, Wang X (2017). Metal-free boron-containing heterogeneous catalysts. Angew Chem Int Ed Engl.

[14] Huang C, Chen C, Zhang M, Lin L, Ye X, Lin S, Antonietti M, Wang X (2015). Carbon-doped BN nanosheets for metal-free photoredox catalysis. Nat Commun.

[15] Zheng M, Cai W, Fang Y, Wang X (2020). Nanoscale boron carbonitride semiconductors for photoredox catalysis. Nanoscale.

[16] Xie YP, Liu G, Lu GQ, Cheng H-M (2012). Boron oxynitride nanoclusters on tungsten trioxide as a metal-free cocatalyst for photocatalytic oxygen evolution from water splitting. Nanoscale.

[17] Bhat S, Wiehl L, Molina-Luna L, Mugnaioli E, Lauterbach S, Sicolo S, Kroll P, Duerrschnabel M, Nishiyama N, Kolb U, Albe K, Kleebe H-J, Riedel R (2015). High-pressure synthesis of novel boron oxynitride B6N4O3 with sphalerite type structure. Chem Mater.

[18] Shi L, Li P, Zhou W, Wang T, Chang K, Zhang H, Kako T, Liu G, Ye J (2016). n-type boron phosphide as a highly stable, metal-free, visible-light-active photocatalyst for hydrogen evolution. Nano Energy.

[19] Sugimoto H, Somogyi B, Nakamura T, Zhou H, Ichihashi Y, Nishiyama S, Gali A, Fujii M (2019). Size-dependent photocatalytic activity of cubic boron phosphide nanocrystals in the quantum confinement regime. J Phys Chem C.

[20] Agnoli S, Favaro M (2016). Doping graphene with boron: a review of synthesis methods, physicochemical characterization, and emerging applications. J Mater Chem A.

[21] Wang Y, Li H, Yao J, Wang X, Antonietti M (2011). Synthesis of boron doped polymeric carbon nitride solids and their use as metal-free catalysts for aliphatic C–H bond oxidation. Chem Sci.

[22] Duan X, Li W, Ao Z, Kang J, Tian W, Zhang H, Ho S-H, Sun H, Wang S (2019). Origins of boron catalysis in peroxymonosulfate activation and advanced oxidation. J Mater Chem A.

[23] Liu G, Meng X, Zhang H, Zhao G, Pang H, Wang T, Li P, Kako T, Ye J (2017). Elemental boron for efficient carbon dioxide reduction under light irradiation. Angew Chem Int Ed Engl.

[24] Wang W-N, Ogi T, Kaihatsu Y, Iskandar F, Okuyama K (2011). Novel rare-earth-free tunable-color-emitting BCNO phosphors. J Mater Chem.

[25] Lei W, Portehault D, Dimova R, Antonietti M (2011). Boron carbon nitride nanostructures from salt melts: tunable water-soluble phosphors. J Am Chem Soc.

[26] Wang J, Ma F, Sun M (2017). Graphene, hexagonal boron nitride, and their heterostructures: properties and applications. RSC Adv.

[27] Single L, Chopra V (2011). Effect of preparation conditions on the crystallinity of chemically synthesized BCNO nanophosphor. J Mater Sci Technol.

[28] Lin L, Ma L, Zhang S, Huang J, Allwood DA (2015). Simple growth of BCNO@C core shell fibres and luminescent BCNO tubes. CrystEngComm.

[29] Ozturk B, de-Luna-Bugallo A, Panaitescu E, Chiaramonti AN, Liu F, Vargas A, Jiang X, Kharche N, Yavuzcetin O, Alnaji M, Ford MJ, Lok J, Zhao Y, King N, Dhar NK, Dubey M, Nayak SK, Sridhar S, Kar S (2015) Atomically thin layers of B–N–C–O with tunable composition Sci Adv 1:e1500094 10.1126/sciadv.1500094 PMC4646774 26601211

[30] Lu F, Zhang X, Lu Z, Xu X, Tang C (2013). Effects of annealing temperature and ambient atmosphere on the structure and photoluminescence of BCNO phosphors. J Lumin.

[31] Zhang X, Yan S, Cheng Y, Gao K, Lu Z, Meng F, Lin J, Xu X, Zhao J, Tang C (2013). Spectral properties of BCNO phosphor with wide range of excitation and emission. Mater Lett.

[32] Zhang X, Lu Z, Liu H, Lin J, Xu X, Meng F, Zhao J, Tang C (2015). Blue emitting BCNO phosphors with high quantum yields. J Mater Chem C.

[33] de Mello JC, Wittmann HF, Friend RH (1997). An improved experimental determination of external photoluminescence quantum efficiency. Adv Mater.

[34] Cui L, Liu Y, Fang X, Yin C, Li S, Sun D, Kang S (2018). Scalable and clean exfoliation of graphitic carbon nitride in NaClO solution: enriched surface active sites for enhanced photocatalytic H2 evolution. Green Chem.

[35] Liu Q, Jiang P, Pu Z, Asiri AM, Al-Youbi AO, Sun X (2014). BCNO nanoparticles: a novel highly efficient fluorosensor for ultrarapid detection of Cu2+. Sens Actuators B Chem.

[36] Ho W, Zhang Z, Xu M, Zhang X, Wang X, Huang Y (2015). Enhanced visible-light-driven photocatalytic removal of NO: Effect on layer distortion on g-C3N4 by H2 heating. Appl Cata B Environ.

[37] Alkoy S, Toy C, Gönül T, Tekin A (1997). Crystallization behavior and characterization of turbostratic boron nitride. J Eur Ceram Soc.

[38] Vieira MA, Gonçalves GR, Cipriano DF, Schettino MA, Silva Filho EA, Cunha AG, Emmerich FG, Freitas JCC (2016). Synthesis of graphite oxide from milled graphite studied by solid-state 13C nuclear magnetic resonance. Carbon.

[39] Li ZQ, Lu CJ, Xia ZP, Zhou Y, Luo Z (2007). X-ray diffraction patterns of graphite and turbostratic carbon. Carbon.

[40] Ebrahim AM, Rodríguez-Castellón E, Montenegro JM, Bandosz TJ (2015). Effect of chemical heterogeneity on photoluminescence of graphite oxide treated with S-/N-containing modifiers. Appl Surf Sci.

[41] Singh M, Kaushal S, Singh P, Sharma J (2018). Boron doped graphene oxide with enhanced photocatalytic activity for organic pollutants. J Photochem Photobiol A.

[42] Ci L, Song L, Jin C, Jariwala D, Wu D, Li Y, Srivastava A, Wang ZF, Storr K, Balicas L, Liu F, Ajayan PM (2010). Atomic layers of hybridized boron nitride and graphene domains. Nat Mater.

[43] Sadhanala HK, Nanda KK (2016). Boron-doped carbon nanoparticles: Size-independent color tunability from red to blue and bioimaging applications. Carbon.

[44] Choi Y, Kang B, Lee J, Kim S, Kim GT, Kang H, Lee BR, Kim H, Shim S-H, Lee G, Kwon O-H, Kim B-S (2016). Integrative approach toward uncovering the origin of photoluminescence in dual heteroatom-doped carbon nanodots. Chem Mater.

[45] Zhu T, Li S, Ren B, Zhang L, Dong L, Tan L (2019). Plasma-induced synthesis of boron and nitrogen co-doped reduced graphene oxide for super-capacitors. J Mater Sci.

[46] Chen S, Li P, Xu S, Pan X, Fu Q, Bao X (2018). Carbon doping of hexagonal boron nitride porous materials toward CO2 capture. J Mater Chem A.

[47] Tang C, Bando Y, Zhi C, Golberg D (2007) Boron–oxygen luminescence centres in boron–nitrogen systems. Chem Commun 4599 10.1039/b711807d 17989804

[48] Örnek M, Hwang C, Reddy KM, Domnich V, Miller SL, Akdoğan EK, Hemker KJ, Haber RA (2018). Formation of BN from BCNO and the development of ordered BN structure: I. Synthesis of BCNO with various chemistries and degrees of crystallinity and reaction mechanism on BN formation. Ceram Int.

[49] Krivanek OL, Chisholm MF, Nicolosi V, Pennycook TJ, Corbin GJ, Dellby N, Murfitt MF, Own CS, Szilagyi ZS, Oxley MP, Pantelides ST, Pennycook SJ (2010). Atom-by-atom structural and chemical analysis by annular dark-field electron microscopy. Nature.

[50] Tran TT, Bray K, Ford MJ, Toth M, Aharonovich I (2016). Quantum emission from hexagonal boron nitride monolayers. Nat Nanotechnol.

[51] Gervais C, Framery E, Duriez C, Maquet J, Vaultier M, Babonneau F (2005). 11B and 15N solid state NMR investigation of a boron nitride preceramic polymer prepared by ammonolysis of borazine. J Eur Ceram Soc.

[52] Dorn RW, Ryan MJ, Kim T-H, Goh TW, Heintz PM, Zhou L, Huang W, Rossini A (2019) Identifying the molecular edge termination of exfoliated hexagonal boron nitride nanosheets with solid-state NMR spectroscopy and plane-wave dft calculations

[53] Ashbrook SE, Duer MJ (2006) Structural information from quadrupolar nuclei in solid state NMR. Concepts Magn Reson Part A 28A:183–248

[54] Kroeker S, Stebbins JF (2001). Three-coordinated boron-11 chemical shifts in borates. Inorg Chem.

[55] Love AM, Thomas B, Specht SE, Hanrahan MP, Venegas JM, Burt SP, Grant JT, Cendejas MC, McDermott WP, Rossini AJ, Hermans I (2019). Probing the transformation of boron nitride catalysts under oxidative dehydrogenation conditions. J Am Chem Soc.

[56] Neiner D, Sevryugina YV, Harrower LS, Schubert DM (2017). Structure and properties of sodium enneaborate, Na2[B8O11(OH)4]·B(OH)3·2H2O. Inorg Chem.

[57] Matsoso BJ, Ranganathan K, Mutuma BK, Lerotholi T, Jones G, Coville NJ (2017). Synthesis and characterization of boron carbon oxynitride films with tunable composition using methane, boric acid and ammonia. New J Chem.

[58] Dong G, Jacobs DL, Zang L, Wang C (2017). Carbon vacancy regulated photoreduction of NO to N2 over ultrathin g-C3N4 nanosheets. Appl Catal B Environ.

[59] Beniwal S, Hooper J, Miller DP, Costa PS, Chen G, Liu S-Y, Dowben PA, Sykes ECH, Zurek E, Enders A (2017). Graphene-like boron–carbon–nitrogen monolayers. ACS Nano.

[60] Yang Q, Wang CB, Zhang S, Zhang DM, Shen Q, Zhang LM (2010). Effect of nitrogen pressure on structure and optical properties of pulsed laser deposited BCN thin films. Surf Coat Technol.

[61] Dontsova D, Fettkenhauer C, Papaefthimiou V, Schmidt J, Antonietti M (2016). 1,2,4-triazole-based approach to noble-metal-free visible-light driven water splitting over carbon nitrides. Chem Mater.

[62] Zhang Y, Mori T, Niu L, Ye J (2011). Non-covalent doping of graphitic carbon nitride polymer with graphene: controlled electronic structure and enhanced optoelectronic conversion. Energy Environ Sci.

[63] Xiang Q, Yu J, Jaroniec M (2011). Preparation and enhanced visible-light photocatalytic H2-production activity of graphene/C3N4 composites. J Phys Chem C.

[64] Prakash K, Karuthapandian S (2021). Construction of novel metal-free graphene oxide/graphitic carbon nitride nanohybrids: a 2D–2D amalgamation for the effective dedyeing of waste water. J Inorg Organomet Polym.

[65] Rahman MZ, Zhang J, Tang Y, Davey K, Qiao S-Z (2017). Graphene oxide coupled carbon nitride homo-heterojunction photocatalyst for enhanced hydrogen production. Mater Chem Front.

[66] Stadie NP, Billeter E, Piveteau L, Kravchyk KV, Döbeli M, Kovalenko MV (2017). Direct synthesis of bulk boron-doped graphitic carbon. Chem Mater.

[67] Weston L, Wickramaratne D, Mackoit M, Alkauskas A, Van de Walle CG (2018). Native point defects and impurities in hexagonal boron nitride. Phys Rev B.

[68] Sajid A, Reimers JR, Ford MJ (2018). Defect states in hexagonal boron nitride: assignments of observed properties and prediction of properties relevant to quantum computation. Phys Rev B.

[69] Katzir A, Suss JT, Zunger A, Halperin A (1975). Point defects in hexagonal boron nitride. I. EPR, thermoluminescence, and thermally-stimulated-current measurements. Phys Rev B.

[70] Lopatin VV, Konusov FV (1992). Energetic states in the boron nitride band gap. J Phys Chem Solids.

[71] Museur L, Feldbach E, Kanaev A (2008). Defect-related photoluminescence of hexagonal boron nitride. Phys Rev B.

[72] Liu X, Ye S, Dong G, Qiao Y, Ruan J, Zhuang Y, Zhang Q, Lin G, Chen D, Qiu J (2009). Spectroscopic investigation on BCNO-based phosphor: photoluminescence and long persistent phosphorescence. J Phys D Appl Phys.

[73] Ren C, Zhang X, Zhou L, Lu Z, Lin J, Xu X, Li L, Zhang X, Xue Y, Meng F, Zhao J, Tang C (2014). Preparation optimization and spectral properties of BCNO phosphors with high quantum efficiency. J Lumin.

[74] Zhang X, Jia X, Liu H, Lu Z, Ma X, Meng F, Zhao J, Tang C (2015). Spectral properties and luminescence mechanism of red emitting BCNO phosphors. RSC Adv.

[75] Strauss V, Margraf JT, Dolle C, Butz B, Nacken TJ, Walter J, Bauer W, Peukert W, Spiecker E, Clark T, Guldi DM (2014). Carbon nanodots: toward a comprehensive understanding of their photoluminescence. J Am Chem Soc.

[76] Li L, Dong T (2018). Photoluminescence tuning in carbon dots: surface passivation or/and functionalization, heteroatom doping. J Mater Chem C.

[77] Eda G, Lin Y-Y, Mattevi C, Yamaguchi H, Chen H-A, Chen I-S, Chen C-W, Chhowalla M (2010). Blue photoluminescence from chemically derived graphene oxide. Adv Mater.

[78] Loh KP, Bao Q, Eda G, Chhowalla M (2010). Graphene oxide as a chemically tunable platform for optical applications. Nat Chem.

[79] Li X, Rui M, Song J, Shen Z, Zeng H (2015). Carbon and graphene quantum dots for optoelectronic and energy devices: a review. Adv Funct Mater.

[80] Wang L, Zhang J, Qu B, Wu Q, Zhou R, Li D, Zhang B, Ren M, Zeng XC (2017). Mechanistic insights into tunable luminescence and persistent luminescence of the full-color-emitting BCNO phosphors. Carbon.

[81] Bao L, Liu C, Zhang Z-L, Pang D-W (2015). Photoluminescence-tunable carbon nanodots: surface-state energy-gap tuning. Adv Mater.

[82] Li X, Lau SP, Tang L, Ji R, Yang P (2014). Sulphur doping: a facile approach to tune the electronic structure and optical properties of graphene quantum dots. Nanoscale.

[83] Qu D, Sun Z, Zheng M, Li J, Zhang Y, Zhang G, Zhao H, Liu X, Xie Z (2015). Three colors emission from S, N Co-doped graphene quantum dots for visible light H2 production and bioimaging. Adv Opt Mater.

[84] Li H, He X, Kang Z, Huang H, Liu Y, Liu J, Lian S, Tsang CHA, Yang X, Lee S-T (2010). Water-soluble fluorescent carbon quantum dots and photocatalyst design. Angew Chem.

[85] Luo Z, Lu Y, Somers LA, Johnson ATC (2009). High yield preparation of macroscopic graphene oxide membranes. J Am Chem Soc.

[86] Lin L, Zhang S (2012). Creating high yield water soluble luminescent graphene quantum dots via exfoliating and disintegrating carbon nanotubes and graphite flakes. Chem Commun.

[87] Yoon H, Chang YH, Song SH, Lee E-S, Jin SH, Park C, Lee J, Kim BH, Kang HJ, Kim Y-H, Jeon S (2016). Intrinsic photoluminescence emission from subdomained graphene quantum dots. Adv Mater.

[88] Peng H, Li Y, Jiang C, Luo C, Qi R, Huang R, Duan C-G, Travas-Sejdic J (2016). Tuning the properties of luminescent nitrogen-doped carbon dots by reaction precursors. Carbon.

[89] Dey S, Govindaraj A, Biswas K, Rao CNR (2014). Luminescence properties of boron and nitrogen doped graphene quantum dots prepared from arc-discharge-generated doped graphene samples. Chem Phys Lett.

[90] Tang L, Ji R, Cao X, Lin J, Jiang H, Li X, Teng KS, Luk CM, Zeng S, Hao J, Lau SP (2012). Deep ultraviolet photoluminescence of water-soluble self-passivated graphene quantum dots. ACS Nano.

[91] Ye C, Li J-X, Li Z-J, Li X-B, Fan X-B, Zhang L-P, Chen B, Tung C-H, Wu L-Z (2015). Enhanced driving force and charge separation efficiency of protonated g-C3N4 for photocatalytic O2 evolution. ACS Catal.

[92] Cowan AJ, Durrant JR (2013). Long-lived charge separated states in nanostructured semiconductor photoelectrodes for the production of solar fuels. Chem Soc Rev.

[93] Maeda K, Domen K (2007). New non-oxide photocatalysts designed for overall water splitting under visible light. J Phys Chem C.

[94] Walsh JJ, Jiang C, Tang J, Cowan AJ (2016). Photochemical CO2 reduction using structurally controlled g-C3N4. Phys Chem Chem Phys.

[95] Lau VW, Moudrakovski I, Botari T, Weinberger S, Mesch MB, Duppel V, Senker J, Blum V, Lotsch BV (2016). Rational design of carbon nitride photocatalysts by identification of cyanamide defects as catalytically relevant sites. Nat Commun.

[96] Sagara N, Kamimura S, Tsubota T, Ohno T (2016). Photoelectrochemical CO2 reduction by a p-type boron-doped g-C3N4 electrode under visible light. Appl Catal B Environ.

[97] Yang L, Jiang S, Zhao Y, Zhu L, Chen S, Wang X, Wu Q, Ma J, Ma Y, Hu Z (2011). Boron-doped carbon nanotubes as metal-free electrocatalysts for the oxygen reduction reaction. Angew Chem Int Ed.

[98] Lei W, Portehault D, Liu D, Qin S, Chen Y (2013). Porous boron nitride nanosheets for effective water cleaning. Nat Commun.

[99] Jiao Y, Zheng Y, Jaroniec M, Qiao SZ (2014). Origin of the electrocatalytic oxygen reduction activity of graphene-based catalysts: a roadmap to achieve the best performance. J Am Chem Soc.

[100] Ferrighi L, Datteo M, Di Valentin C (2014). Boosting graphene reactivity with oxygen by boron doping: density functional theory modeling of the reaction path. J Phys Chem C.

[101] Ajmal A, Majeed I, Malik RN, Idriss H, Nadeem MA (2014). Principles and mechanisms of photocatalytic dye degradation on TiO2 based photocatalysts: a comparative overview. RSC Adv.

[102] Dante RC, Martín-Ramos P, Chamorro-Posada P, Meejoo-Smith S, Vázquez-Cabo J, Rubiños-López Ó, Lartundo-Rojas L, Sánchez-Árevalo FM, Trakulmututa J, Rutto D, Deebansok S, Srikhaow A (2019). Comparison of the activities of C2N and BCNO towards Congo red degradation. Mater Chem Phys.

