# Supplementary material for: Boron Carbon Oxynitride as a Novel Metal-Free Photocatalyst
Source: Nanoscale Res Lett. 2021 Dec 11;16:176. doi: 10.1186/s11671-021-03629-5 (PMC8665969; doi:10.1186/s11671-021-03629-5)
Supplement: Supplementary file 1 — Additional file 1. Table S1: Different sets of precursors used in the preparation of BCNO. Table S2: List of experiment and BCNO sample names investigated in this study. Figure S1: TEM images of (a) BGH01LT, (b)BGH01-30min, (c) BGH01-6hr, and (d) BGH02 in low magnification, (e) BGH02 in high magnification, (f) BGH03 in low magnification, (g) BGH03 in high magnification. (h) EDS mapping analysis of BGH03 (1) B (2) C (3) N (4) O. (i) BGH01LT, (j) BGH01-30min, (k) BGH01, and (l) BGH03 under UV lamp (365 nm, 4 watts). Figure S2: TEM images of (a) BMH01-30min, (b)BMH01HT, (c) BMH02. (d) EDS mapping analysis of BMH03. (1) B (2) C (3) N (4) O. (e) BMH01-30min (f) BMH01HT (g) BMH01 and (h) BMH03 under UV lamp (365 nm, 4 watts). Figure S3: Stacked XRD of (i) BGH01LT, (ii) BGH02, (iii) BGH03, (iv) BMH01-30min, (v) BMH01HT, (vi) BMH02. Figure S4: (a) Stacked XRD (normalized intensity) of g-C3N4, BMH01, and the physical mixture of both. (b) Stacked XRD (normalized intensity) of g-C3N4, BGH01, and the physical mixture of both. Table S3: XPS surface elemental composition BGH01, BGH03 and BMH01, BMH03. Figure S5: (a) Stacked FTIR of BGH01 (blue trace), BGH03 (red trace) and BGH01LT (green trace) and their corresponding IR active functional groups. (b) Stacked FTIR of BMH01 (blue trace), BMH03 (pink trace) and BMH01-30min (orange trace) and their corresponding IR active functional groups. Figure S6: Evolution of bonding composition, B-N, B-C and B-O in BGH series with reaction temperature and time based on XPS analyses. Figure S7: (a) Survey XPS spectra of BMH03, and 11B NMR spectroscopies. Core level spectra of (b) B 1s, (c) N 1s, (d) C 1s, (e) O 1s. Each core spectra were fitted with a black trace, while the red and green traces under the peak were deconvoluted using a Gaussian function. (f) 11B solid state MAS NMR were further deconvoluted with topspin SOLA software. Figure S9: Evolution of tetracoordinate boron site for BMH series based on 11B solid state MAS NMR analysis. Figu [file 11671_2021_3629_MOESM1_ESM.docx]

**Supplementary Information**

**Boron Carbon Oxynitride as a novel metal-free photocatalyst**

Liang Cheng Chien^a^,‡, Chen Wei Chiang^a^,‡, Chou Chio Lao^a^, Yung-I Lin^a^, Hao-Wu Lin^a^, Pei Yuin Keng.^a,^*

Department of Materials Science and Engineering, National Tsing Hua University, Hsinchu City, 30013, Taiwan

‡ Authors contributed equally

Corresponding author email: Keng.py@gapp.nthu.edu.tw

Optimization of BCNO synthesis

| Sources | Precursor 1 | Precursor 2 |
| --- | --- | --- |
| B | Boric acid | Boric acid |
| C | Hexamethylenetetramine | Hexamethylenetetramine |
| N | Guanidine hydrochloride | Melamine |
|  |  |  |

**Table S1:** Different sets of precursors used in the preparation of BCNO.

| Precursor | Temperature | Time (hr) | Mole ratio (B:N:C) | Sample name |
| --- | --- | --- | --- | --- |
| 1 (boric acid, guanidine hydrochloride, hexamethylenetetramine) | 600 °C | 12 | 3:1:0.1 | BGH01LT |
|  | 800 °C | 0.5 | 3:1:0.1 | BGH01-30min |
|  | 800 °C | 6 | 3:1:0.1 | BGH01-6hr |
|  | 800 °C | 12 | 3:1:0.1 | BGH01 |
|  | 800 °C | 12 | 3:1:0.2 | BGH02 |
|  | 800 °C | 12 | 3:1:0.3 | BGH03 |
| 2 (boric acid, melamine, hexamethylenetetramine) | 600 °C | 12 | 3:1:0.1 | BMH01 |
|  | 800 °C | 0.5 | 3:1:0.1 | BMH01-30min |
|  | 800 °C | 12 | 3:1:0.1 | BMH01HT |
|  | 600 °C | 12 | 3:1:0.2 | BMH02 |
|  | 600 °C | 12 | 3:1:0.2 | BMH03 |

**Table S2:** List of experiment and BCNO sample names investigated in this study.

**Figure S1:** TEM images of (a) BGH01LT, (b)BGH01-30min, (c) BGH01-6hr, (d) BGH02 in low magnification, (e) BGH02 in high magnification, (f) BGH03 in low magnification, (g) BGH03 in high magnification. (h) EDS mapping analysis of BGH03 (1) B (2) C (3) N (4) O. (i) BGH01LT, (j) BGH01-30min, (k) BGH01, and (l) BGH03 under UV lamp (365 nm, 4 watts).


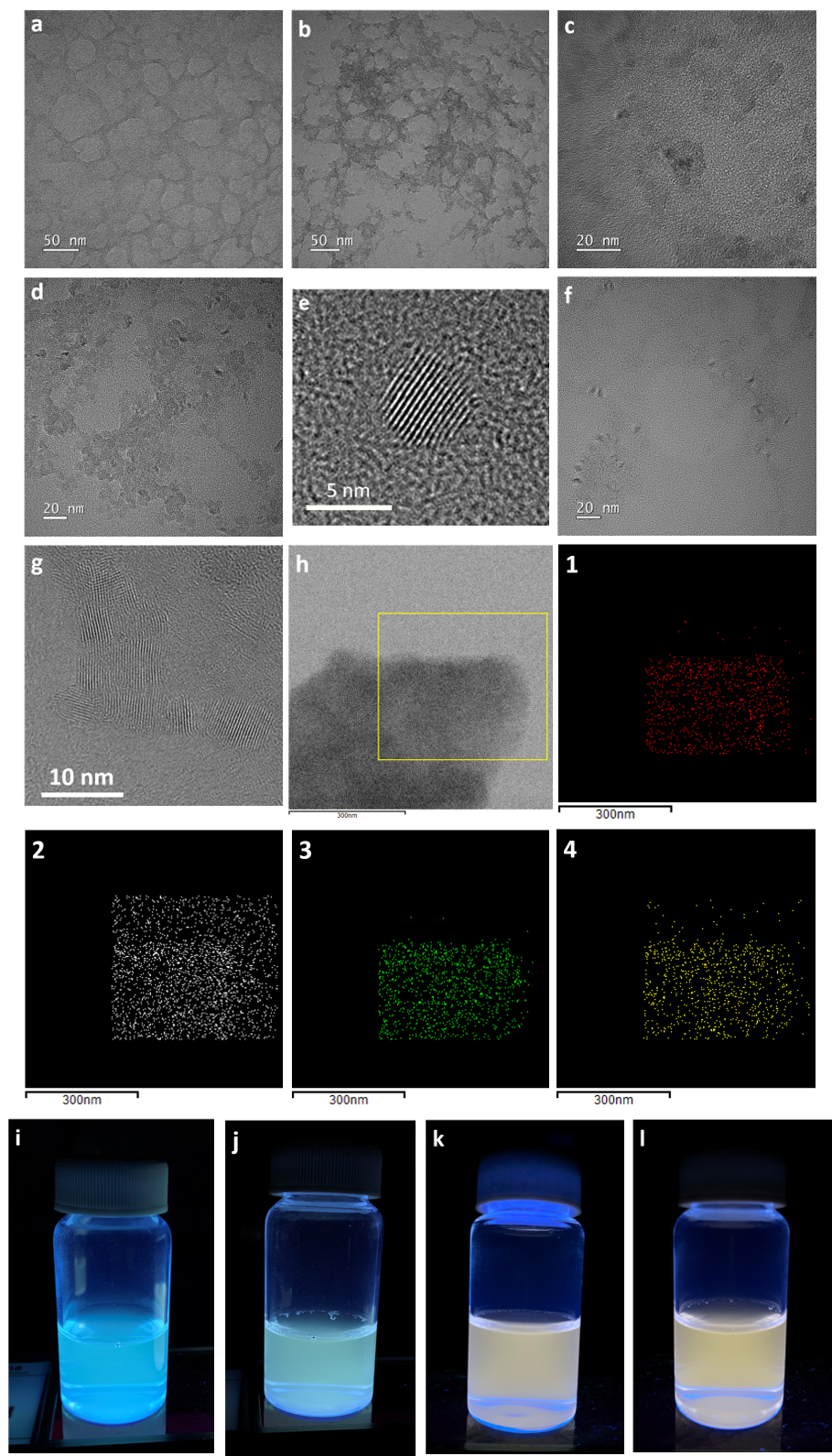


**Figure S2:** TEM images of (a) BMH01-30min, (b)BMH01HT, (c) BMH02. (d) EDS mapping analysis of BMH03. (1) B (2) C (3) N (4) O. (e) BMH01-30min (f) BMH01HT (g) BMH01 and (h) BMH03 under UV lamp (365 nm, 4 watts).


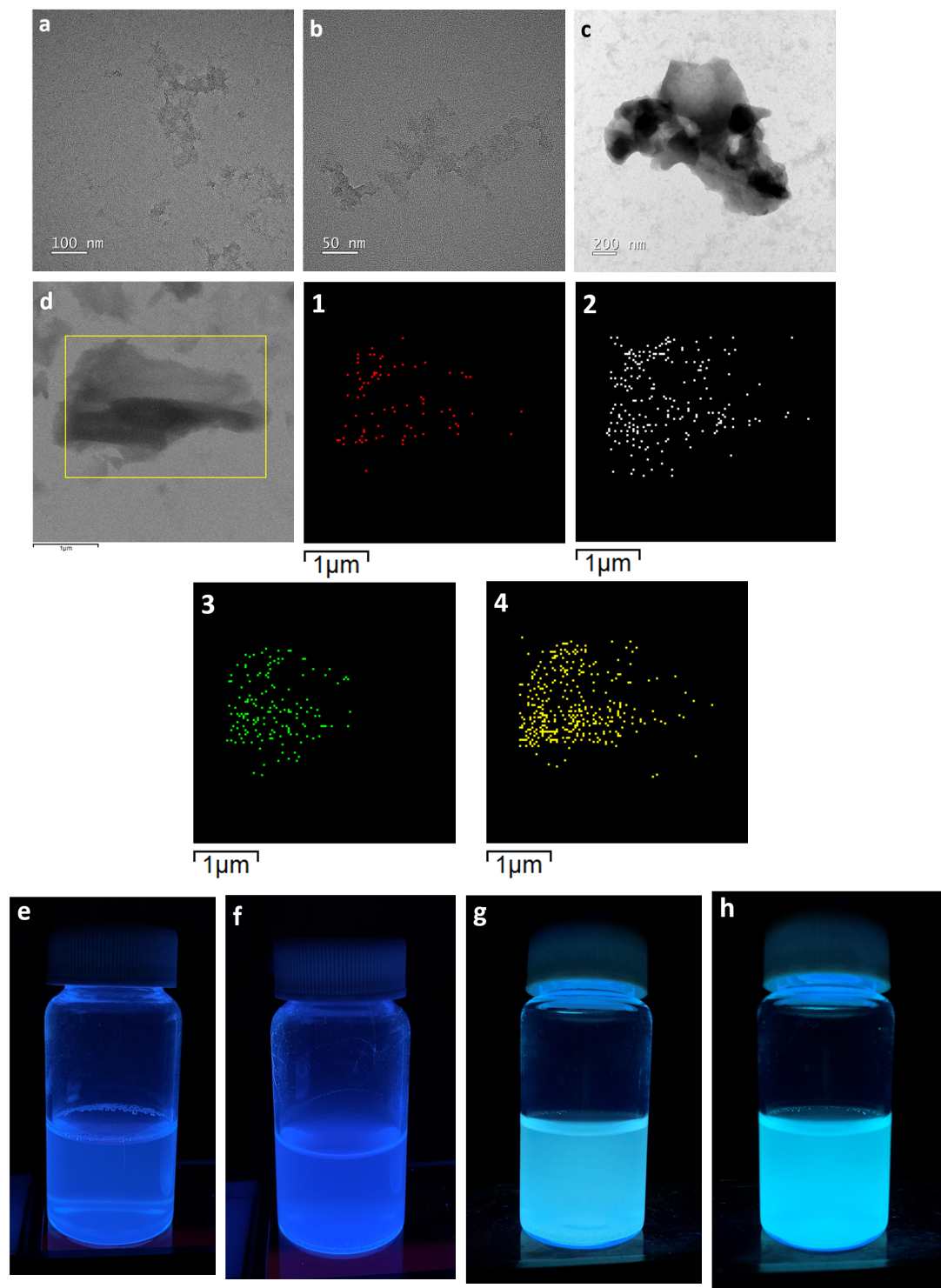


Figure S1 and S2 showed representative TEM images of BCNO nanostructure annealed with different precursor under different conditions. For BGH series, BGH01LT annealed at 600℃ for 12 hours and BGH01-30min annealed at 800℃ for 30 minutes both exhibited a honeycomb morphology, due to the incomplete formation of BN structure (Fig. S1(a) and (b)). Upon increasing temperature and heating time, BGH01-6hr, BGH02, and BGH03 all possessed crystalline core with disk-like morphology as shown in Figure S1(c), (d) and (f). The crystalline disk shaped BGH02 (Fig. 1(e)), and BGH03 (Fig. 1(g)) is measured at their longest axis to be 7 ±2 nm and 6.6 ±1 nm, respectively. For BMH series, BMH01HT-30min annealed at 800 ℃ for 30 minutes, BMH01HT annealed at 800℃ for 12 hours and BMH02 all adopted multi-layer nanosheets with low crystallinity and ill-defined shape (Figure S2).


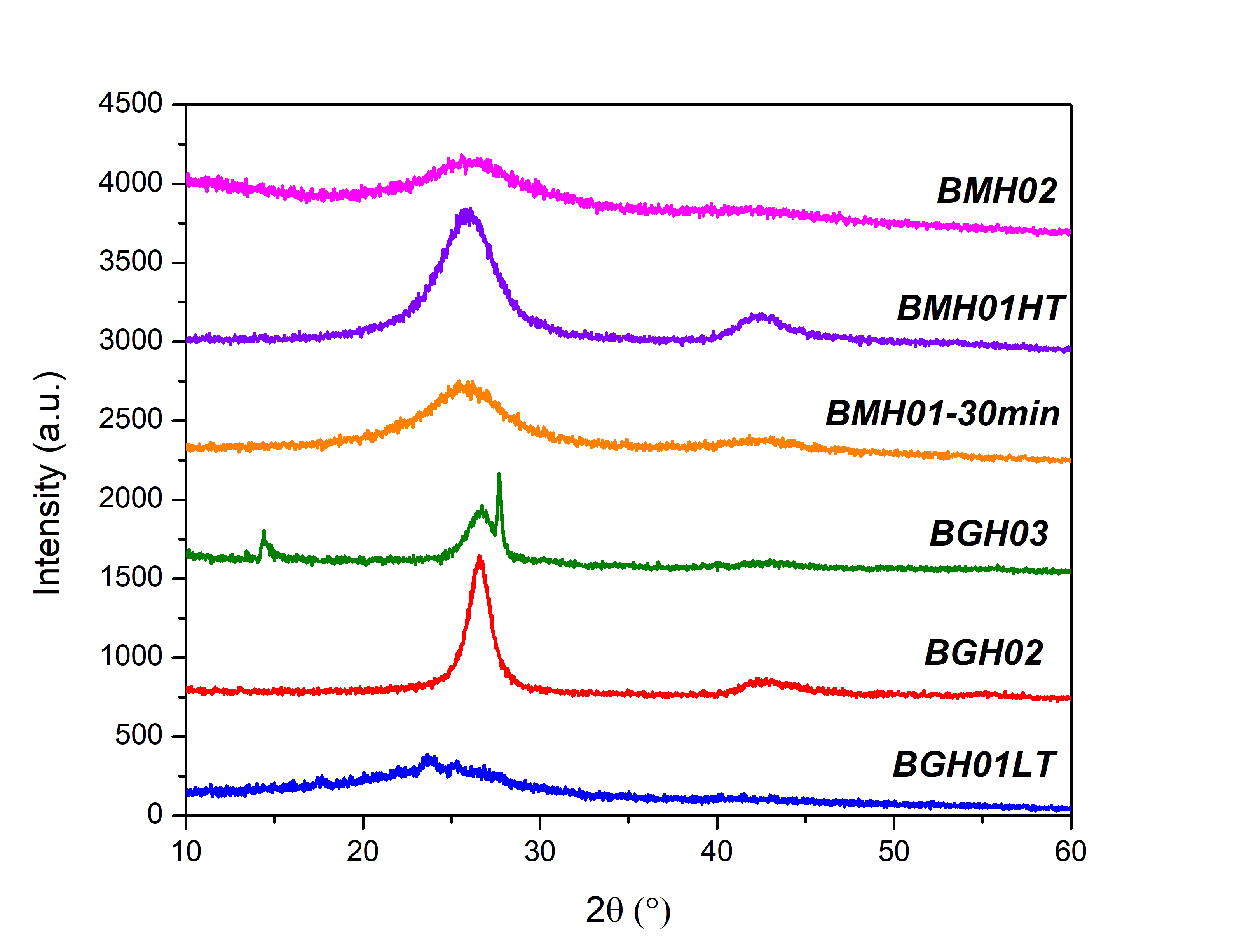


**Figure S3:** Stacked XRD of (i) BGH01LT, (ii) BGH02, (iii) BGH03, (iv) BMH01-30min, (v) BMH01HT, (vi) BMH02.

Figure S3 showed XRD patterns of BCNO prepared at a prescribed reaction condition in Table S2. For BMH series, samples synthesized at lower temperature (600°C) calcination exhibited broad characteristic peaks around 25° and 42°, which are denoted as (002) plane reflection and (10) plane reflection of hexagonal crystal structure of graphite, respectively. However, as heating temperature and time increased, the peak around 25° became sharper and shifted to 26°, which suggested the structural transformation from graphite to turbostratic boron nitride. This structural transformation upon increasing calcination temperature is also supported by XPS, FTIR and solid-state NMR analyses. For BGH series, BGH02 and BGH03 showed broad peaks around 26° and 42°, which were denoted as (002) plane reflection and (10) plane reflection of turbostratic boron nitride, respectively. The broadening was induced by the effect of Scherrer equation, which suggested the morphology of extremely fine particles shown in Figure S1. The sharp peaks of BGH03 at 14.4° and 27.7° is attributed to the presence of residual boron oxide.


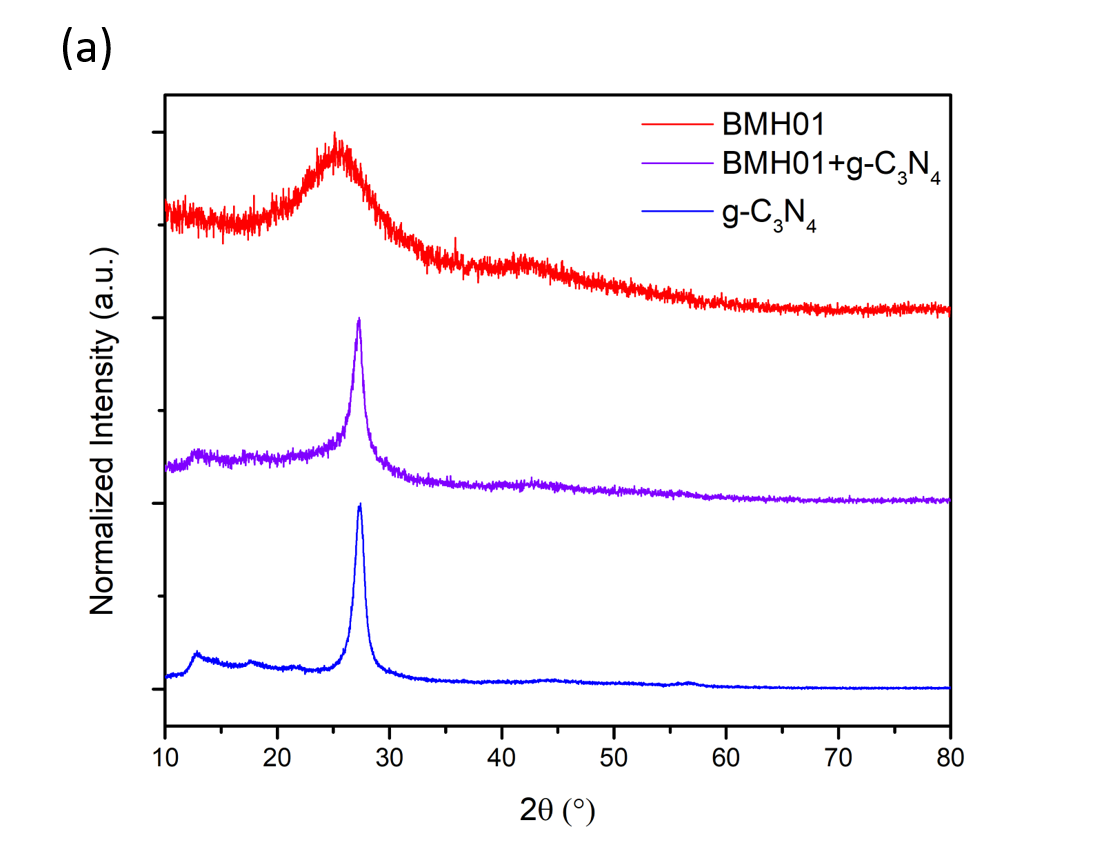

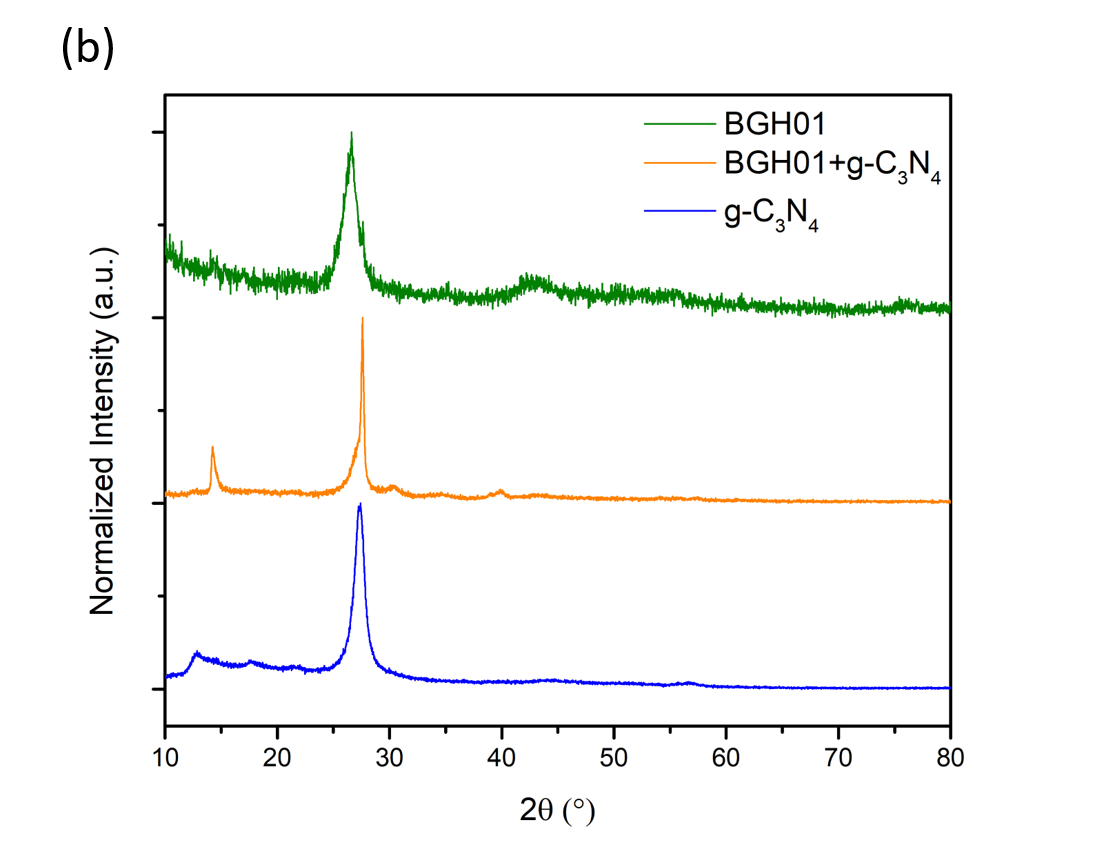


**Figure S4:** (a) Stacked XRD (normalized intensity) of g-C_3_N_4_, BMH01, and the physical mixture of both. (b) Stacked XRD (normalized intensity) of g-C_3_N_4_, BGH01, and the physical mixture of both.


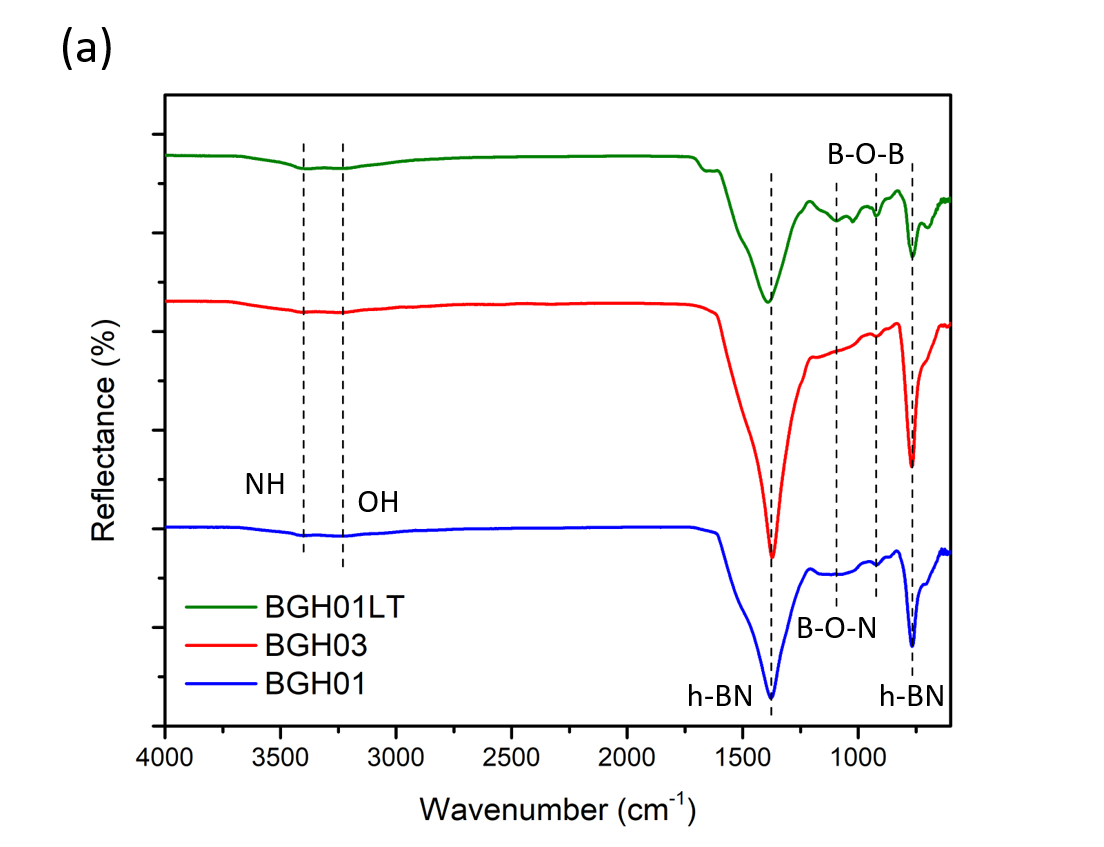

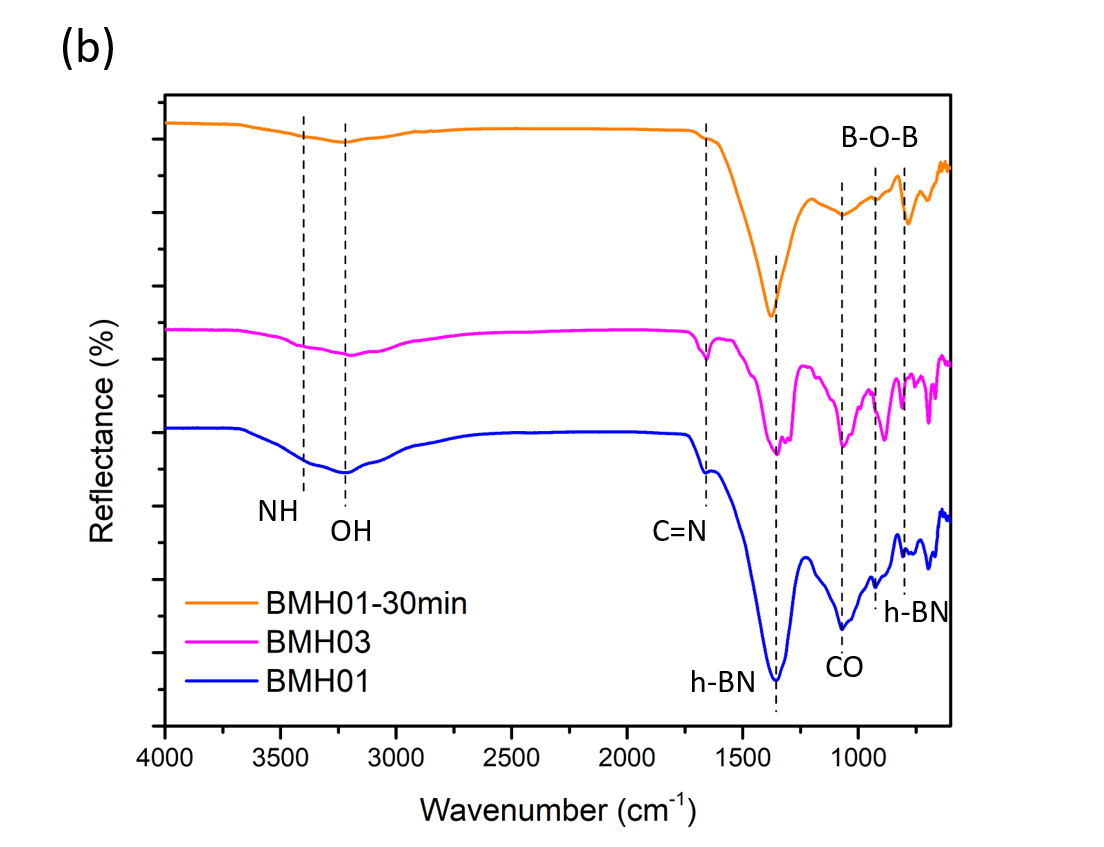


**Figure S5:** (a) Stacked FTIR of BGH01 (blue trace), BGH03 (red trace) and BGH01LT (green trace) and their corresponding IR active functional groups. (b) Stacked FTIR of BMH01 (blue trace), BMH03 (pink trace) and BMH01-30min (orange trace) and their corresponding IR active functional groups.

Fourier transform infrared (FTIR) spectrum of the BGH01 and BMH01 is shown in Figure. S5. In both samples, the presence of a weak and broad N-H and O-H stretching band at ~ 3400 cm^-1^ and ~ 3200 cm^-1^ which is related to the presence of moisture under atmospheric condition. BGH series (Fig. S5(a)) showed two characteristic BN bands at ~ 1376 cm^-1^ and 766 cm^-1^, which corresponded to B-N stretching and B-N-B vibration mode, respectively [1,2]. Commensurate with XPS and 11B NMR, the presence of B-O related functional groups for BGH series can be visualized at ~ 1094 cm^-1^ and 922 cm^-1^. A stacked FTIR for BMH series is shown in Figure. S5(b) showing signature functional groups of C-O stretching at ~ 1070 cm^-1^ in graphite oxides. According to XPS and solid-state NMR analysis, BMH series composed of B, N co-doped graphite oxide along with B-doped carbon nitride and BCNO domain. The presence of nitride (C=N) functional group at ~ 1658 cm^-1^ in BMH01 and BMH03 synthesized at low temperature corroborates with structural analysis. The C=N nitrile bond disappeared as reaction temperature is increased from 600℃ to 800℃ for the BMH01-30min (Fig. S5(b) orange trace), which indicated complete nitration into the BN network at higher calcination temperature. Solid state NMR analysis along with XPS elemental analysis both confirmed that the transformation of B, N co-doped graphite oxides into BN related structures upon increasing calcination temperature. Furthermore, at lower calcination temperature, the appearance of h-BN signature modes and B-O-B bonding at ~ 926 cm-1 in BMH showed successful doping of B and N into the carbon network.

**Table S3:** XPS surface elemental composition BGH01, BGH03 and BMH01, BMH03.


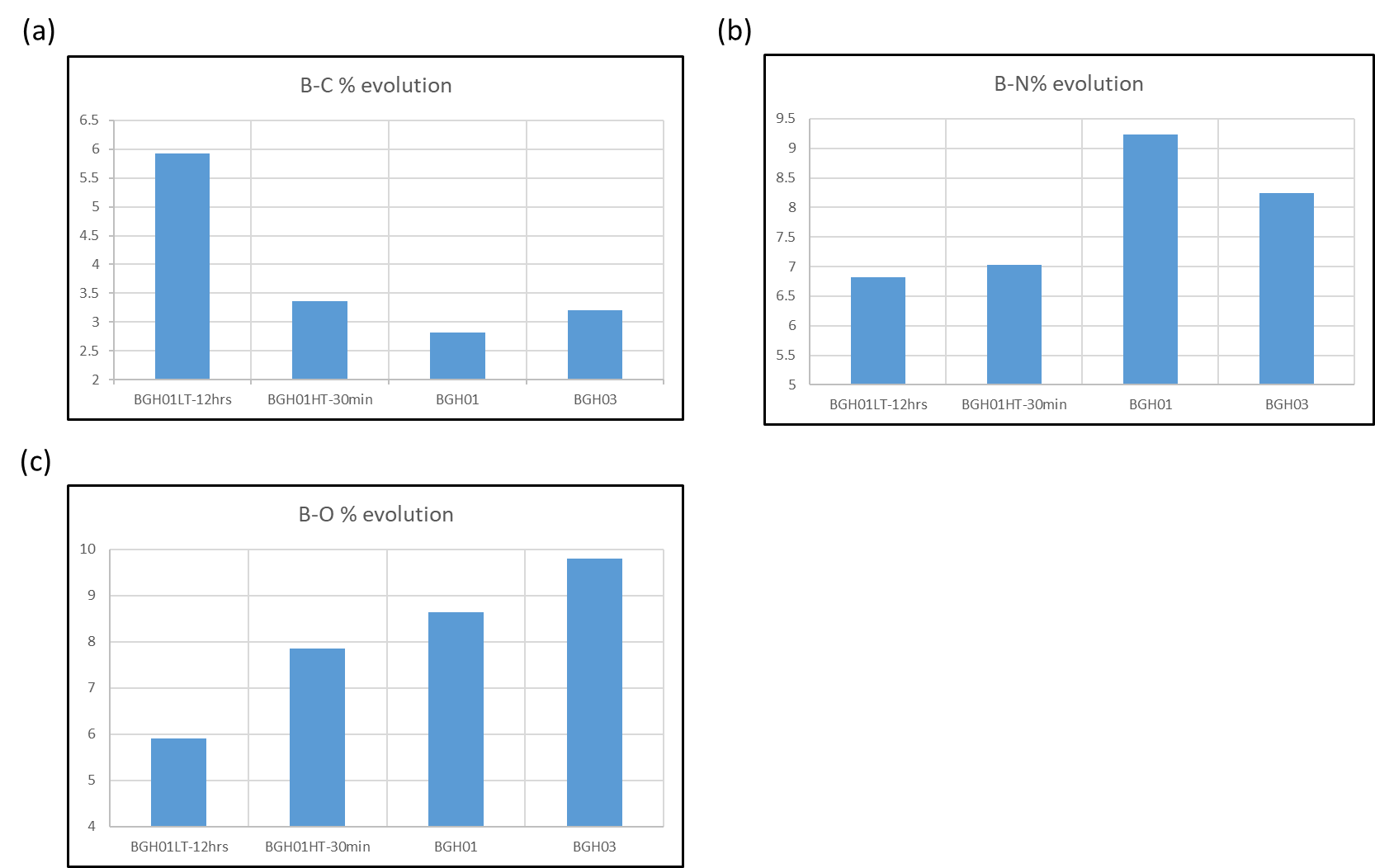


|  | B% | C% | N% | O% |
| --- | --- | --- | --- | --- |
| BGH01 | 40.6 | 7.95 | 37.7 | 13.8 |
| BGH03 | 16.1 | 10.6 | 43.0 | 30.3 |
| BGH01LT-12hrs | 35.6 | 18.18 | 31.4 | 18.9 |
| BMH01 | 5.24 | 75.1 | 9.27 | 10.4 |
| BMH03 | 6.64 | 70.0 | 9.53 | 13.8 |
| BMH01HT-30min | 11.5 | 64.1 | 12.7 | 11.7 |
| BMH01HT-12hrs | 39.1 | 9.71 | 37.3 | 13.9 |

**Figure S6:** Evolution of bonding composition, B-N, B-C and B-O in BGH series with reaction temperature and time based on XPS analyses.

XPS analysis of increase carbon source of BMH03 shows that BMH03 consist of a high composition of C carbon species, while the B1s signal is very weak. The deconvoluted B1s spectra shows that BMH03 have new binding energy peak at around 191 eV, which was assign as B-C-N. The percentage of the C=C bonding increased. This result suggested that increasing in carbon source ratio leads to the increased in graphitic domain.


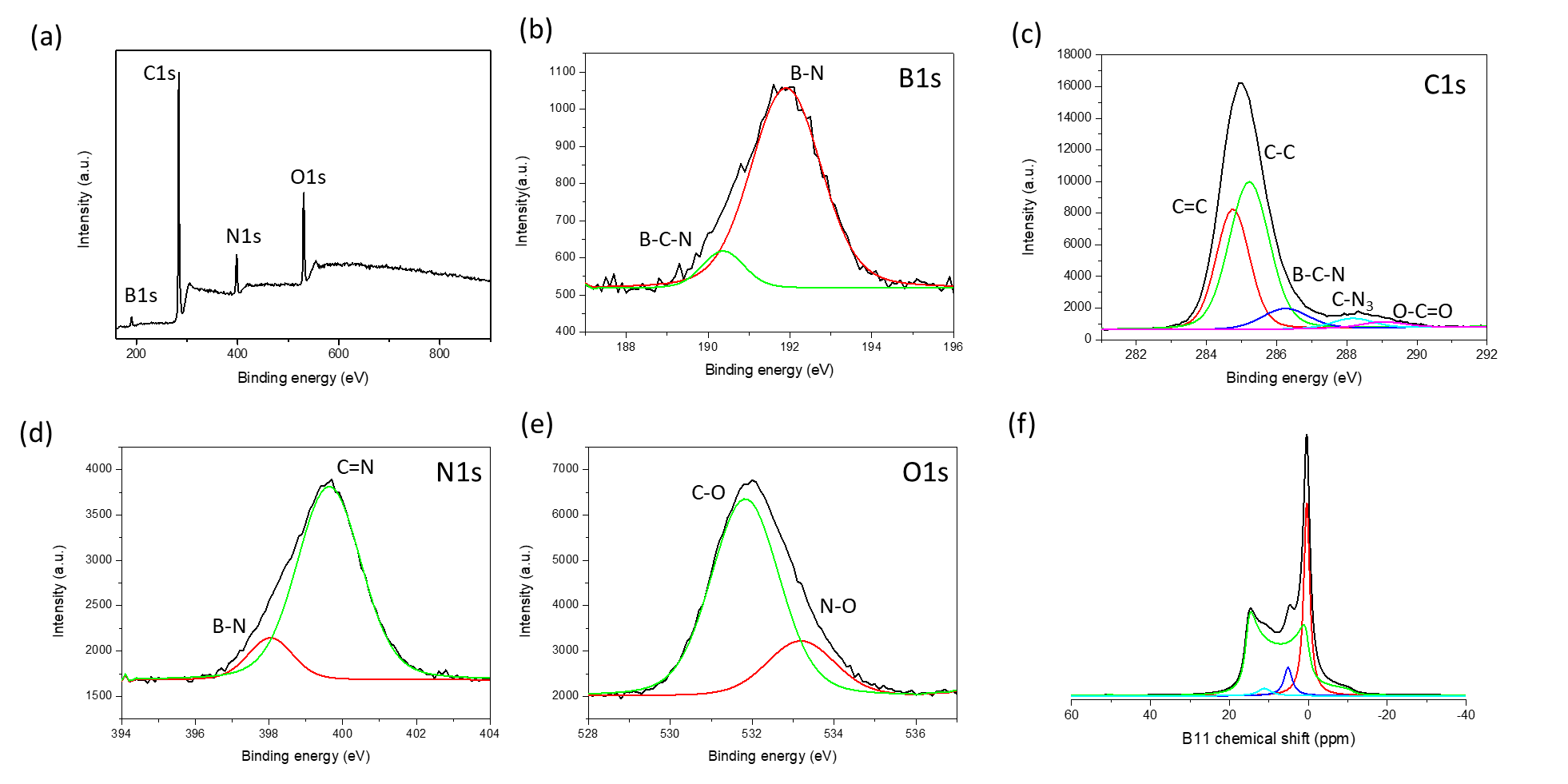


**Figure S7:** (a) Survey XPS spectra of BMH03, and ^11^B NMR spectroscopies. Core level spectra of (b) B 1s, (c) N 1s, (d) C 1s, (e) O 1s. Each core spectra were fitted with a black trace, while the red and green traces under the peak were deconvoluted using a Gaussian function. (f) ^11^B solid state MAS NMR were further deconvoluted with topspin SOLA software.

**Figure S8:** Evolution of tetracoordinate boron site for BGH series based on ^11^B solid state MAS NMR analysis.

Figure S9: Evolution of tetracoordinate boron site for BMH series based on ^11^B solid state MAS NMR analysis.

**Figure S10:** Solid-state ^11^B NMR for BGH01LT showing a high composition of tetracoordinated BN_2_(OH)_2_ bonding, along with the high composition of boron oxides.


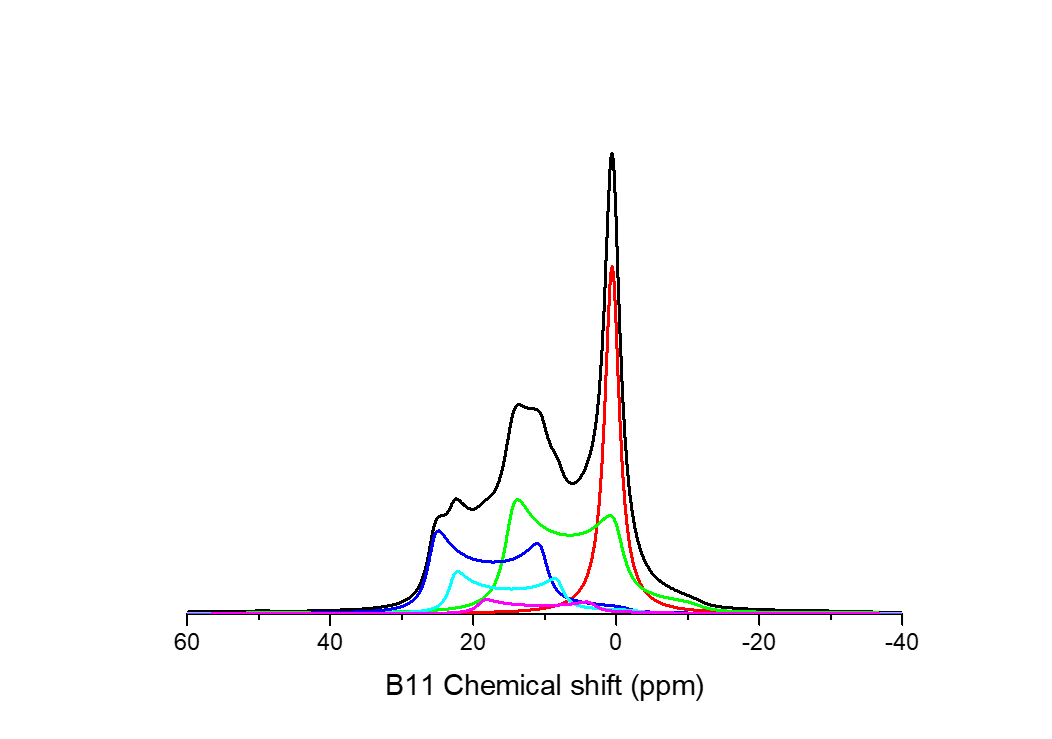


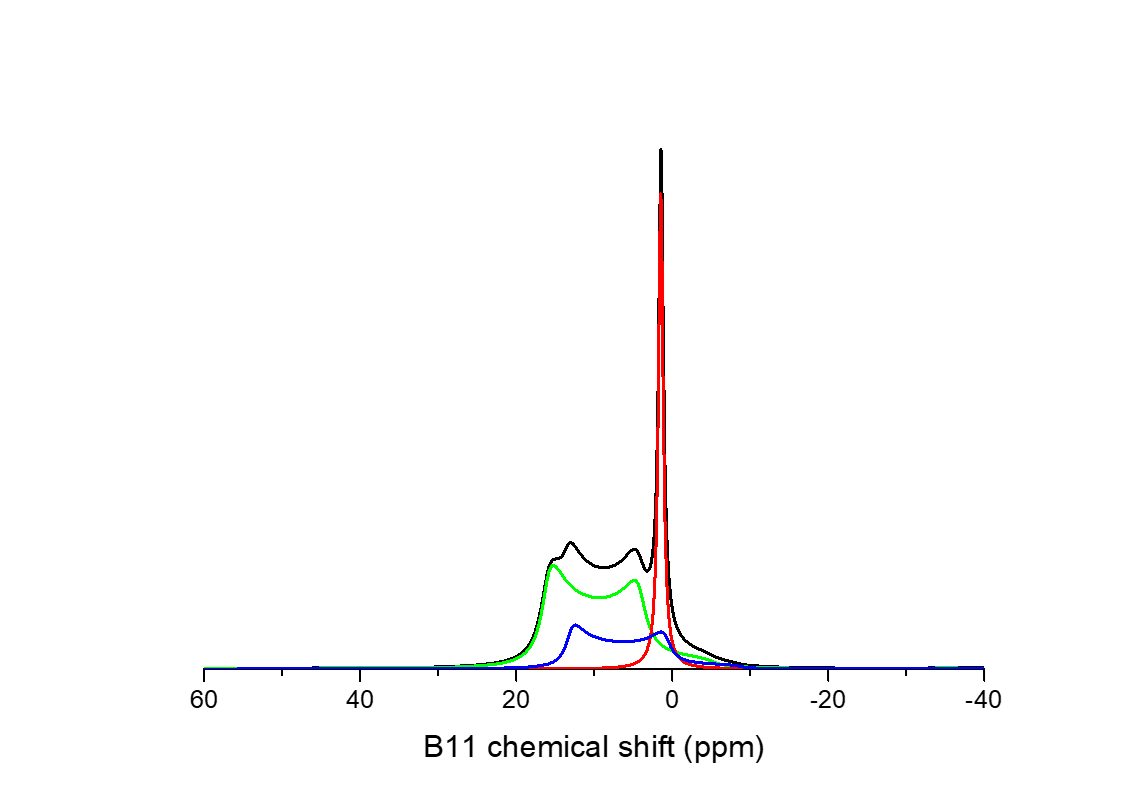


**Figure S11**: Solid-state ^11^B NMR for BMH01-30min showing emergence of BN_3_ and its corresponding oxides. The tetracoordinate boron species here changed from BN_2_(OH)(CO) or BN_2_(CO)_2_ to BN_2_(OH)_2_, which resembles the tetracoordinated B-sites in BGH series.

**Figure S12:** Solid-state B-11 NMR for BMH01HT showing emergence of BN_3_ and its corresponding oxides. The tetracoordinate boron species here changed from BN_2_(OH)(CO) or BN_2_(CO)_2_ to BN_2_(OH)_2_, which resembles the tetracoordinated B-sites in BGH series.


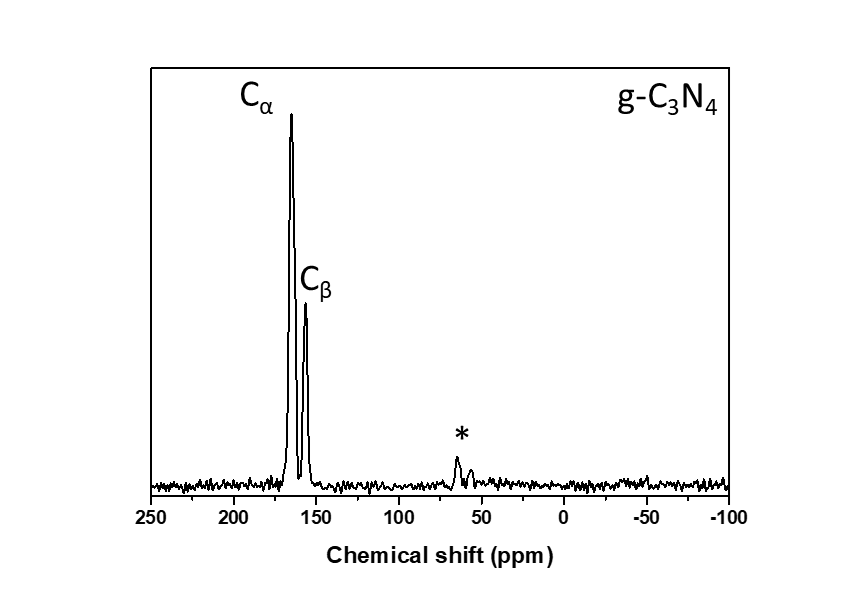


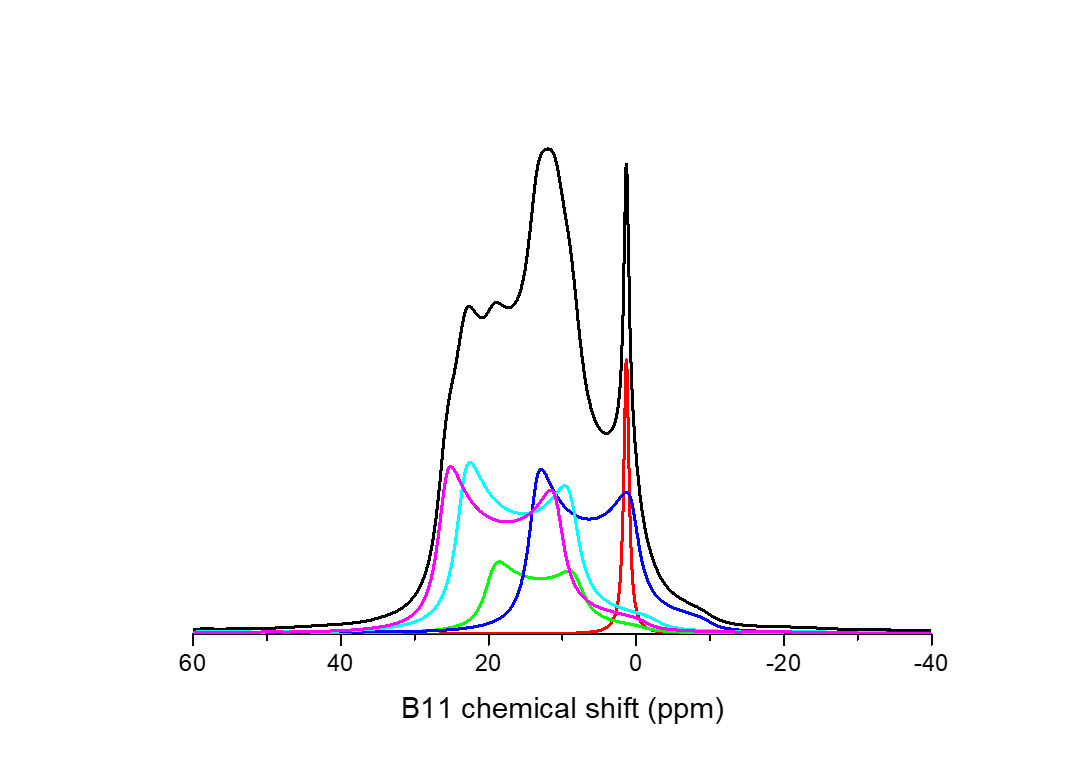


**Figure S13:** Solid state ^13^C MAS NMR of g-C_3_N_4_.

|  | BMH01  Carbon composition | BMH03  Carbon composition |
| --- | --- | --- |
| Cα | 34.6% | 29.4% |
| Cβ | 31.7% | 25.2% |
| graphite | 33.7% | 45.4% |

**Table S4:** Carbon composition of BMH01 and BMH03 based on CP-MAS ^13^C NMR deconvolution data and their corresponding integration values under each peak.

**Figure S14:** Overlay emission spectra of BCNO prepared in this study. (a) Stacked normalized PL spectra and (b) Stacked normalized UV-visible absorbance spectra BMH and BGH series prepared in this study. Excitation wavelength was set at 365 nm. (c) Overlay Tauc plot (αhv)^1/2^ versus h*v* for BMH01 (red trace) and BGH03 (blue trace).


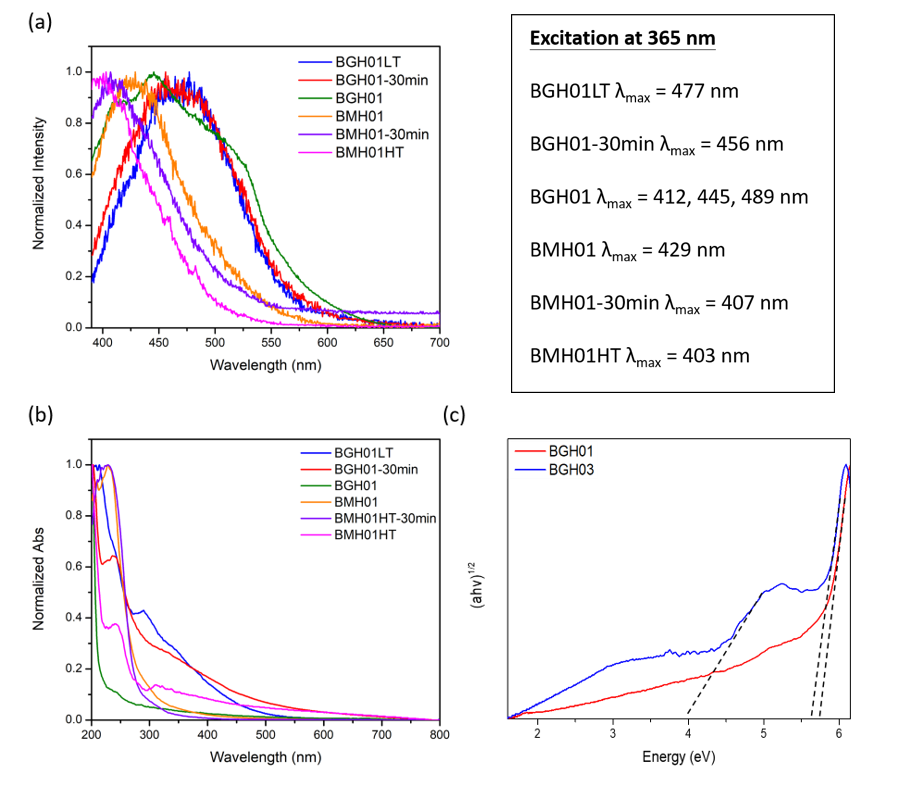


| Sample name | Quantum yield % | I_1_ | τ_1_ | I_2_ | τ_2_ | I_3_ | τ_3_ | τ average (µs) |
| --- | --- | --- | --- | --- | --- | --- | --- | --- |
| BGH01 | 8.4 | 0.984 | 1.00 | 0.0143 | 21.8 | 2.29x10-3 | 126 | 1.58 |
| BGH03 | 9.7 | 0.971 | 1.15 | 0.026 | 20.3 | 3.44x10-3 | 133 | 2.10 |
| BMH01 | 7.6 | 0.907 | 1.53 | 0.082 | 25.5 | 1.39x10-2 | 124 | 5.18 |
| BMH03 | 9.6 | 0.818 | 1.99 | 0.165 | 24.7 | 2.15x10-2 | 115 | 8.14 |

**Table S5:** Quantum yield and multiexponential decay fitting results of the µs-time resolved photoluminescence spectra monitored at λmax of each sample. Excitation wavelength was set at 337 nm.


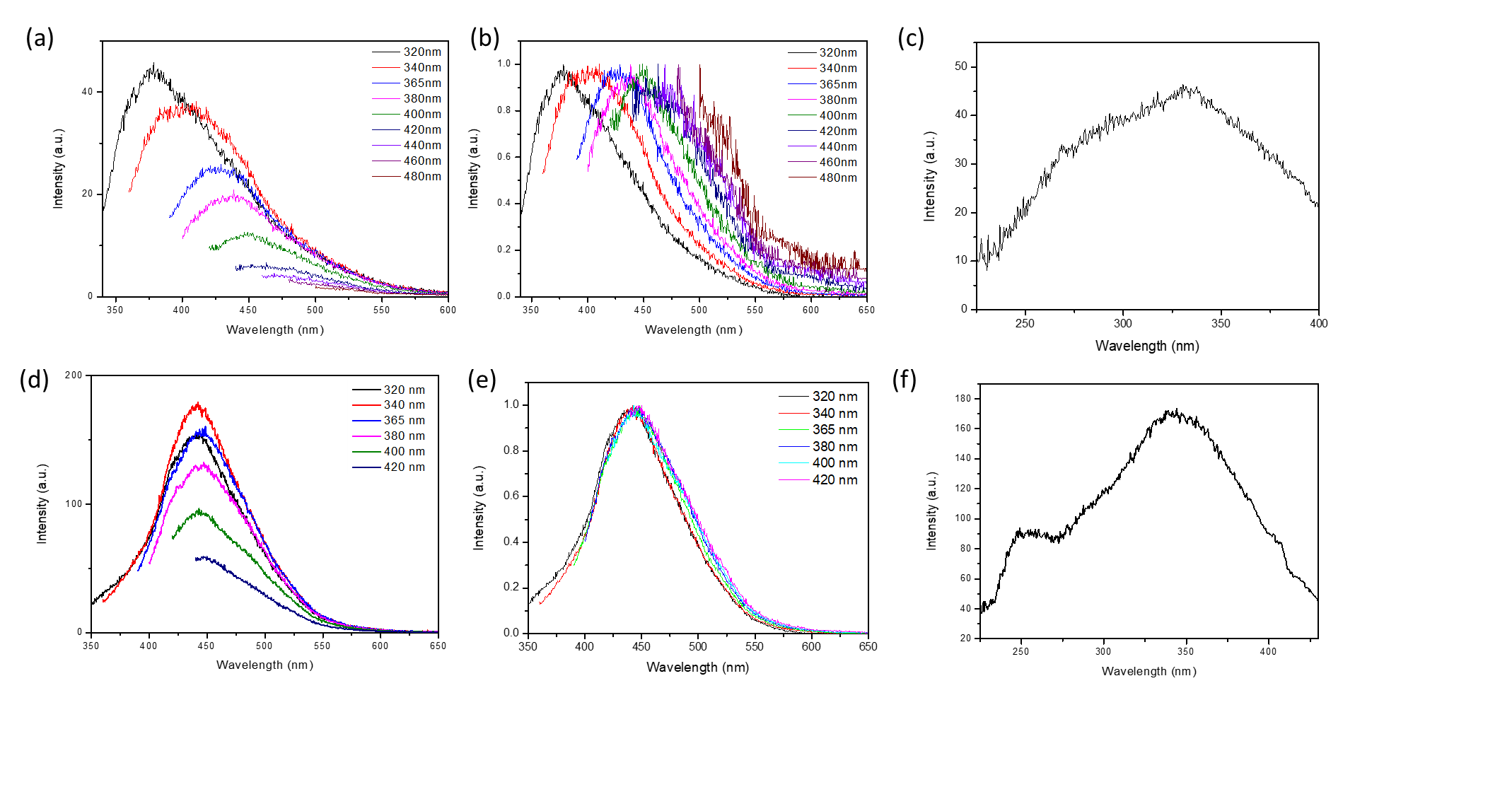


**Figure S15:** Photoluminescence emission of BMH01 and BMH03 at different excitation wavelength. The highest emission intensity is excited with 320 nm light for BMH01 and 340 nm for BMH03. (a) excitation dependent PL spectrum of BMH01 (b) normalized excitation dependent PL spectrum for BMH01 (c) PLE spectrum of BMH01 at 420 nm emission (d) excitation dependent PL spectrum of BMH03 (e) normalized excitation dependent PL spectrum for BMH03 (f) PLE spectrum of BMH03 at 440 nm emission.

**Figure S16:** UV-visible absorbance of methylene blue during dye photodegradation using BMH01-30min.


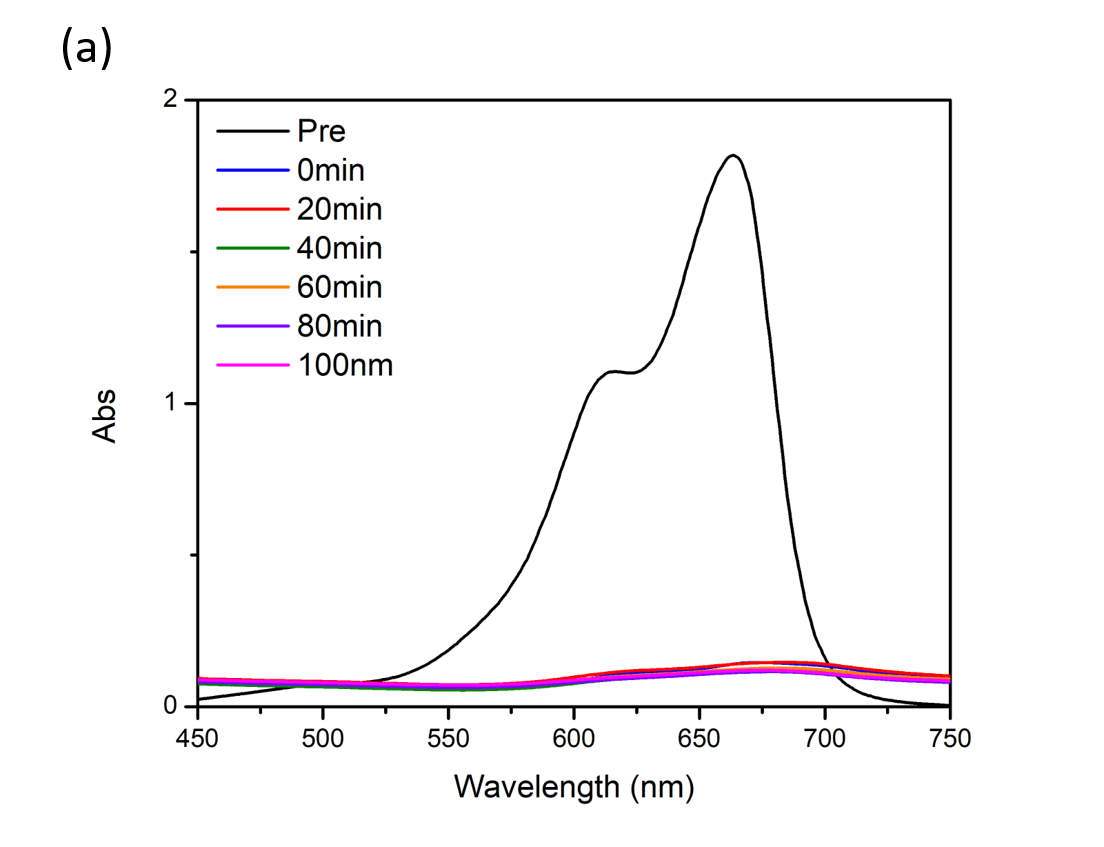

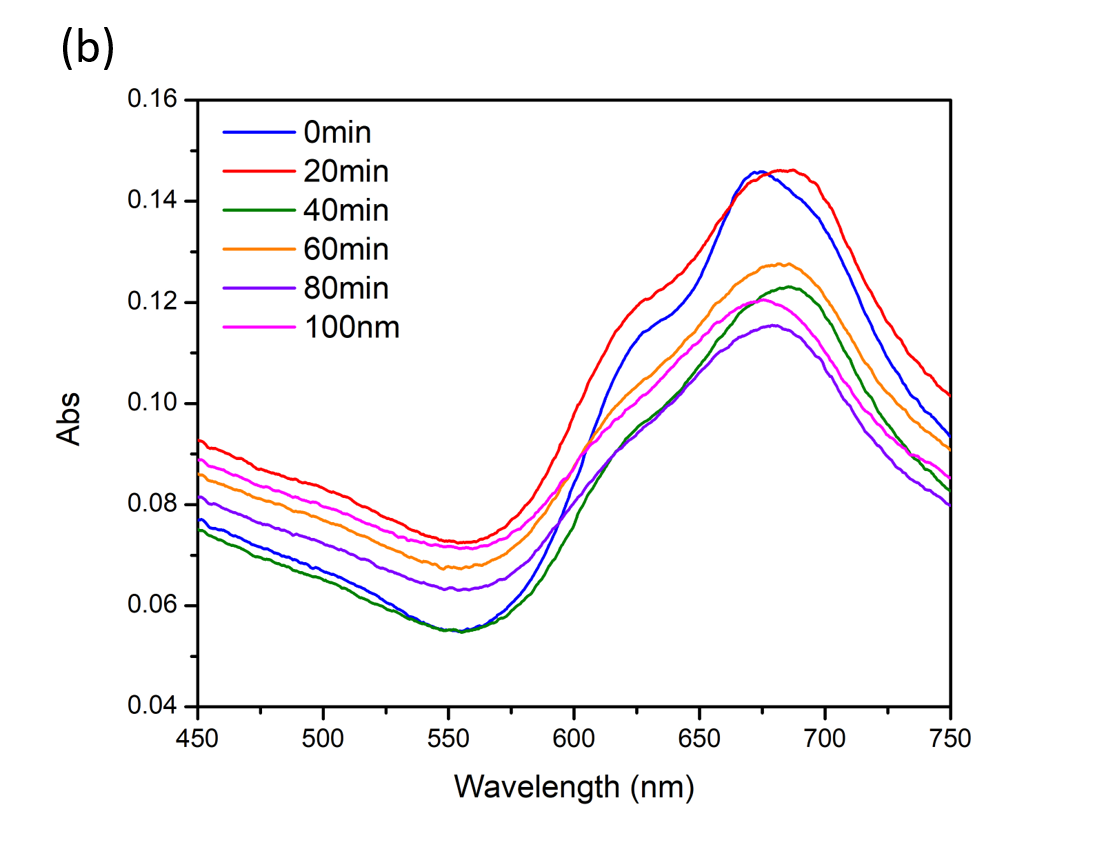


Figure S16 showed a representative dye degradation experiment result using BMH01-30min as photocatalyst under the prescribed photodegradation reaction. In this experiment, BMH01-30min only exhibited high dye adsorption during equilibration in dark. Upon light irradiation, the concentration of dye remained constant throughout the entire 40 minutes of the experiment. BMH01-30 min, BMH01HT, BGH01LT and BGH01-30min also exhibited similar results, in which there is high reduction in dye concentration after the equilibration period in dark. However, upon light irradiation, no further degradation was observed. Thus, we concluded that these samples are photocatalytic inactive despite exhibiting similar optical properties as other BMH and BGH samples. These results confirm that the photocatalytic activity of BCNO is highly correlated with their structures, which is depended on the precursor sources, and reaction conditions. The sharp drop in the absorption of MB form left figure is caused by physical adsorption of MB onto the photocatalyst. The adsorption percentage values of MB are 92.3% for BMH01-30min, 85% for BMH01HT and 52.7% for BGH01-30min, respectively.

**Figure S17:** UPS spectra of (a) BMH01, (b) BMH02, and (c) BMH03.


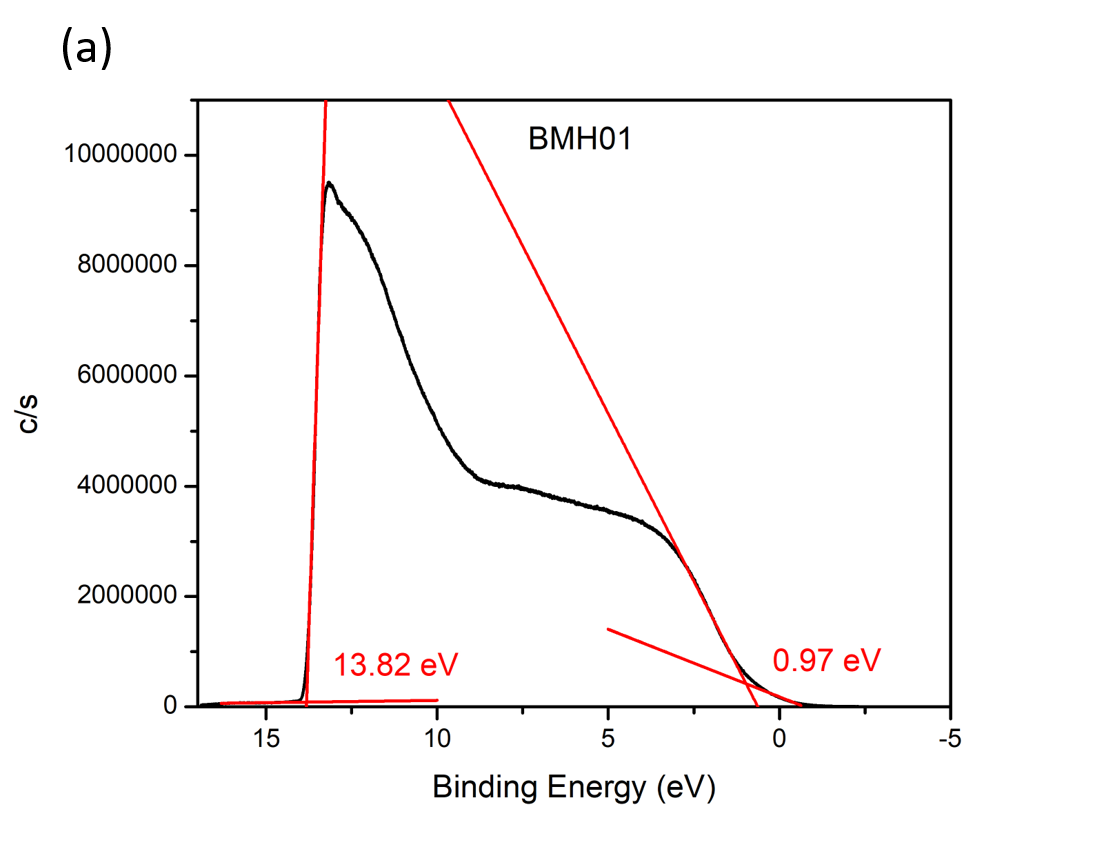

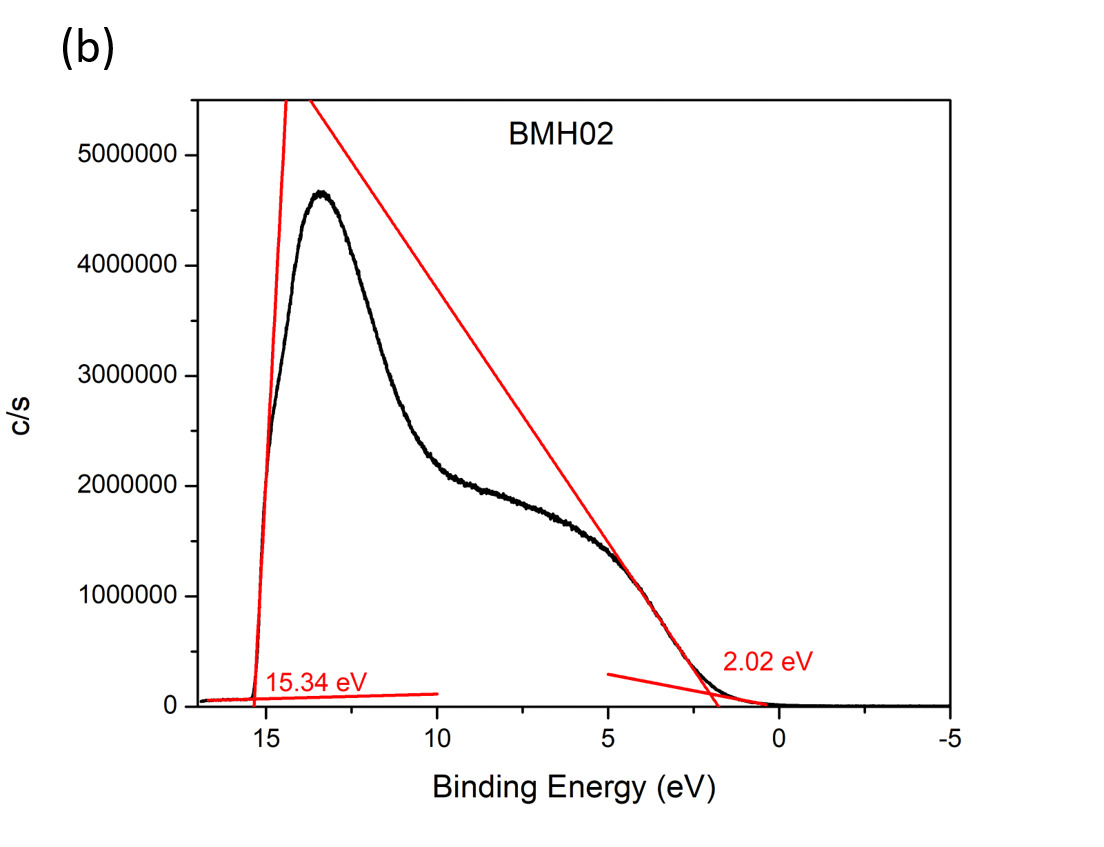


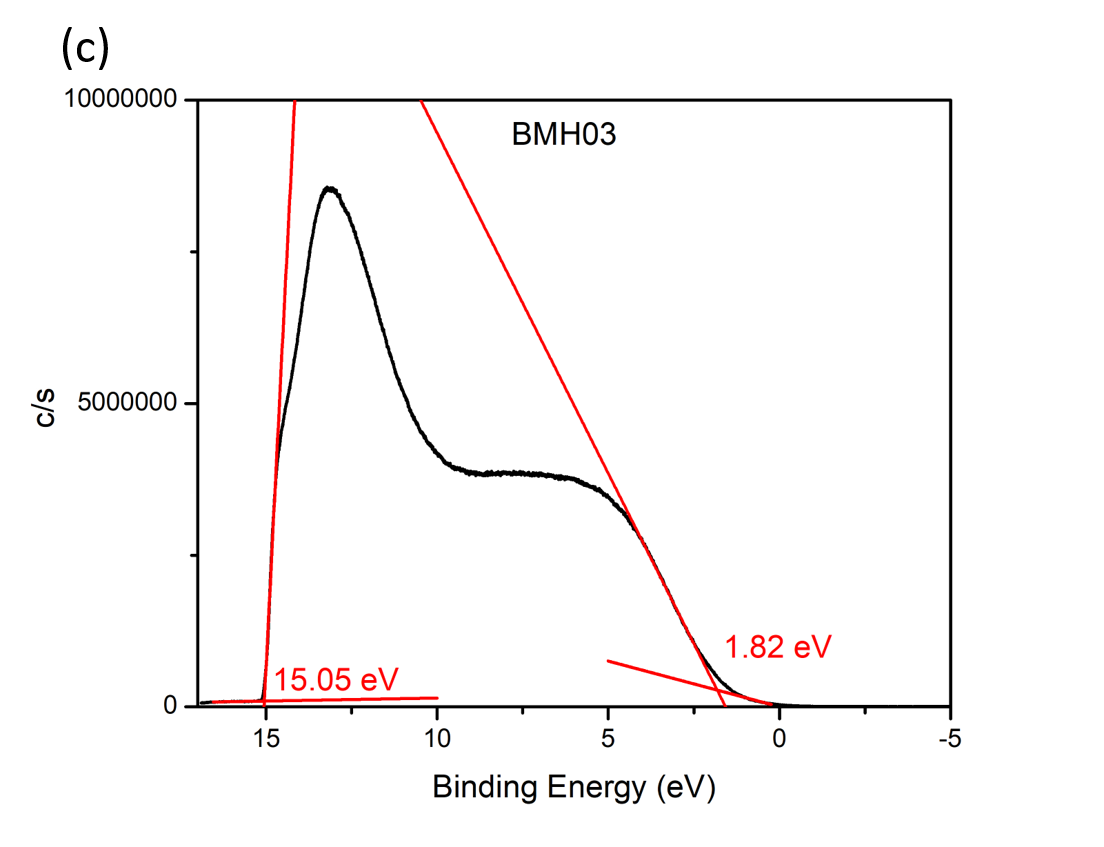


UPS measurement were performed under ultrahigh vacuum in a ULVAC-PHI PHI 5000 Versaprobe II using He I 21.22 eV as a photon source with 5 V bias. The energies of vacuum levels were deduced using the secondary electron cutoff of UPS spectra at normal emission with sample biased by 5 V. Based on UPS analysis, the valence band position of BMH01, BMH02 and BMH03 is presented in the Table S6.

| Sample name | Valence band position vs vacuum (eV) | Valence band position vs NHE (eV) |
| --- | --- | --- |
| BMH01 | -8.4 | 3.9 |
| BMH02 | -7.9 | 3.4 |
| BMH03 | -8 | 3.5 |

**Table S6:** Energy band position of BMH series.

References

[1] Geick R, Perry C H and Rupprecht G 1966 Normal Modes in Hexagonal Boron Nitride *Phys. Rev.* **146** 543–7

[2] Lu F, Zhang X, Lu Z, Xu X and Tang C 2013 Effects of annealing temperature and ambient atmosphere on the structure and photoluminescence of BCNO phosphors *Journal of Luminescence* **143** 343–8
